# Unlocking Solid-State Organometallic Photochemistry with Optically Transparent, Porous Salt Thin Films

**DOI:** 10.1021/jacs.3c09188

**Published:** 2023-11-08

**Authors:** Aishanee Sur, Joe D. Simmons, Andrew A. Ezazi, Kyle J. Korman, Subham Sarkar, Ethan T. Iverson, Eric D. Bloch, David C. Powers

**Affiliations:** † Department of Chemistry, Texas A&M University, College Station, Texas 77843, United States; ∇ Department of Chemistry, Indiana University, Bloomington, Indiana 47405, United States

## Abstract

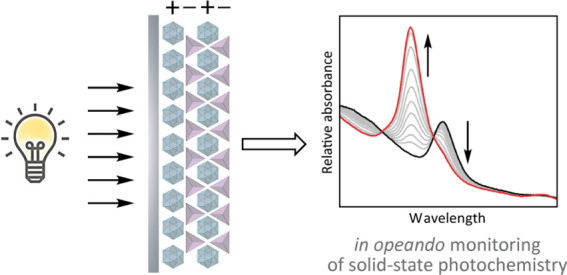

Synthetic porous materials continue to garner attention as platforms for solid-state chemistry and as designer heterogeneous catalysts. Applications in photochemistry and photocatalysis, however, are plagued by poor light harvesting efficiency due to light scattering resulting from sample microcrystallinity and poor optical penetration that arises from inner filter effects. Here we demonstrate the layer-by-layer growth of optically transparent, photochemically active thin films of porous salts. Films are grown by sequential deposition of cationic Zr-based porous coordination cages and anionic Mn porphyrins. Photolysis facilitates the efficient reduction of Mn(III) sites to Mn(II) sites, which can be observed in real-time by transmission UV-vis spectroscopy. Film porosity enables substrate access to the Mn(II) sites and facilitates reversible O_2_ activation in the solid state. These results establish optically transparent, porous salt thin films as versatile platforms for solid-state photochemistry and *in operando* spectroscopy.

Synthetic porous materials, such as metal-organic frameworks (MOFs),^1^ covalent organic frameworks (COFs),^2^ and porous coordination polymers (PCPs),^3^ are conceptually well-suited for application in solid-state photochemistry and photocatalysis.^4^ Reticular synthetic strategies enable systematic variation of the optical density,^5^ absorption profile,^6^ and three-dimensional orientation of chromophores with respect to pore-localized donors or acceptors.^7,8^ In practice, application of synthetic porous materials in photocatalysis is plagued not only by the mass transport and material stability concerns that confront all porous materials in catalysis^9^ but also by inefficient light harvesting. Light scattering caused by the microstructure of solid porous materials (Figure 1a),^10^ along with limited optical penetration due to inner filter effects,^11^ collectively hinder light harvesting in many materials (Figure 1b). For example, assuming Beers Law behavior extends to the solid state, a crystal of tetracarboxyphenylporphyrin (H_2_tcpp) would be expected to absorb 99% of 419 nm photons within 34 nm of the crystallite surface.^12^ Consequently, porous solids are typically characterized by diffuse reflectance spectroscopy, a technique limited to surface phenomena.^13^ Transmission spectroscopy, which offers insight into bulk properties, is unavailable.

**Figure 1:**
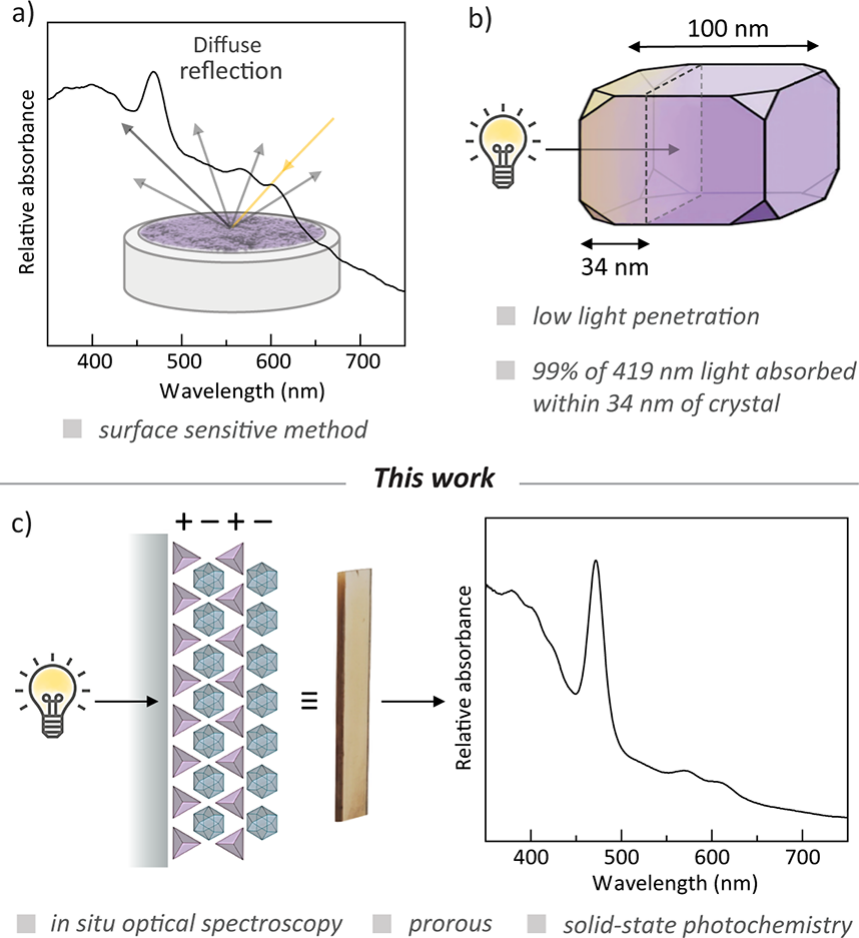
(a) Light scattering by microcrystalline powders mandates characterization by diffuse reflectance spectroscopy. (b) Inner filter effects limit photon penetration in molecular crystals. (c) Here, LbL assembly of optically transparent, porous salt thin films enables solid-state photochemistry and *in situ* optical spectroscopy.

Access to monolithic, optically transparent, porous films would obviate challenges associated with light scattering. Synthetic control over the thickness of such a film would enable the optimization of photon absorption and thus light harvesting efficiency. Existing strategies to generate films of porous materials via solvothermal deposition,^14,15^ template-assisted methods,^16^ vapor phase deposition,^17^ and layer-by-layer (LbL) assembly^18,19^ do not provide a general platform to control composition and structure. For example, while solvothermal deposition and template-assisted synthesis have been applied to metal-organic layers (MOLs)^20^ with ultrathin morphology,^21^ available methods do not provide general access to target materials based on specific chromophores. Given the extended structures of MOFs and MOLs, the absorbing molecules must either be a structural component of the material or postsynthetically incorporated into isolated films.^22^

Here, we demonstrate LbL assembly of monolithic, optically transparent, porous salt thin films based on zirconium coordination cages and Mn-porphyrin-derived anions (Figure 1c). Porous salts are an emerging class of materials assembled by combination of charged permanently porous coordination cages with complementarily charged cages or organometallic complexes.^23−26^ Because porous salts are assembled via Coulombic interactions, not discrete coordination bonds, the cage component can be varied independently of the chromophore structure, which enables the independent optimization of materials and optical properties. In addition, the structural and hydrolytic stabilities of the salt components can be tuned for a given application. We leverage the new thin films for solid-state photochemistry: Photoreduction of Mn(III) to Mn(II) can be observed by *in operando* spectroscopy. Because the films are porous, the resulting Mn(II) films engage in solid-state O_2_ activation. In comparison, a microcrystalline sample of the same porous salt could not be photoreduced, which underscores the challenges inherent in solid-state photochemistry. These findings demonstrate porous thin films as new platforms for realizing and investigating photochemistry within porous solids.

The development of optically transparent porous thin films was guided by several important design criteria. First, we targeted synthetically modular molecular scaffolds that engage in predictable photochemistry for the nonporous, photoactive component. We were attracted by metalloporphyrins, which display strong optical absorbances, are easily tunable by metal-ion substitution or porphyrin functionalization, and are common structural elements in MOFsand catalysts.^27^ Second, to achieve film porosity, we sought optically transparent porous cages with complementary charge. We selected Zr-based cages because these (1) do not absorb in the visible spectrum, (2) display broad chemical and thermal stability, (3) are available in an array of geometries and charge states, (4) exhibit permanent porosity, and (5) have an overall charge of 4^+^ which is complementary to the 4^–^ porphyrin anions.^28^

Based on these concepts, we prepared new porous salts comprised of **[Mn(tcpp)Cl]**^**4–**^ and either [Zr_12_(μ_3_-O)_4_(μ_2_-OH)_12_(Cp)_12_(FDC)_6_]^4+^ (**[ZrFDC]**^**4+**^) or [Zr_12_(μ_3_-O)_4_(μ_2_-OH)_12_(Cp)_12_(Me_2_BDC)_6_]^4+^ (**[ZrMe**_**2**_**BDC]**^**4+**^) (Cp^–^ = cyclopentadienyl; FDC^2–^ = 2,5-furandicarboxylate; Me_2_BDC^2–^ = 2,5-dimethyl-1,4-benzenedicarboxylate). Metathetical combination of **[HNEt**_**3**_**]**_**4**_**[Mn(tcpp)Cl]**, prepared by treatment of H_4_[Mn(tcpp)Cl] with triethylamine (Et_3_N), with porous cage **[ZrFDC]OTf**_**4**_ or **[ZrMe**_**2**_**BDC]OTf**_**4**_ afforded greenish brown precipitates in nearly quantitative yield assuming 1:1 cage:porphyrin, which is consistent with the +4 and −4 charges of the two components. The solids, which were isolated via filtration and washed extensively with MeOH, are poorly crystalline as determined by PXRD (Figure S1 and S2). ^1^H and ^19^F NMR analysis of the supernatant following salt metathesis displays resonances of [HNEt_3_]OTf, the soluble product of salt metathesis, and confirms the absence of cyclopentadiene or cyclopentadienyl fragments, which would be expected from ligand exchange to afford an extended network structure (Figure S3). IR spectra of the isolated solids displayed bands characteristic of both cage and metallopophyrin components (Figures S4 and S5), diffuse reflectance spectra displayed features expected of the porphyrin Soret bands (Figures S6 and 7), and XPS analysis of the solids indicated 1:1 cage:porphyrin (Figures S8 and 9) based on the ratio of Zr:Mn and confirms the absence of OTf^–^ in the solid. The porous salts display high thermal stability as judged by TGA with negligible mass loss up to 300 °C under an N_2_ flow.

After solvent exchange and activation, (**[ZrFDC][Mn(tcpp)Cl]**) and (**[ZrMe**_**2**_**BDC][Mn(tcpp)Cl]**) displayed N_2_-accessible BET (Langmuir) surface areas (77 K) of 333 (564) and 228 (410) m^2^/g, respectively (Figure 2). Due to slow equilibration with N_2_, we typically evaluated porosity by CO_2_ uptake at 195 K; under these conditions surface areas of 135 (244) and 140 (324) m^2^/g were measured. These surface areas are consistent with those observed for previously reported porous salts.^23−25^ Importantly, salts of **[Mn(tcpp)Cl]**^**4–**^ with nonporous counter cations, such as [HNEt_3_]^+^, are not porous under any condition we attempted.

**Figure 2:**
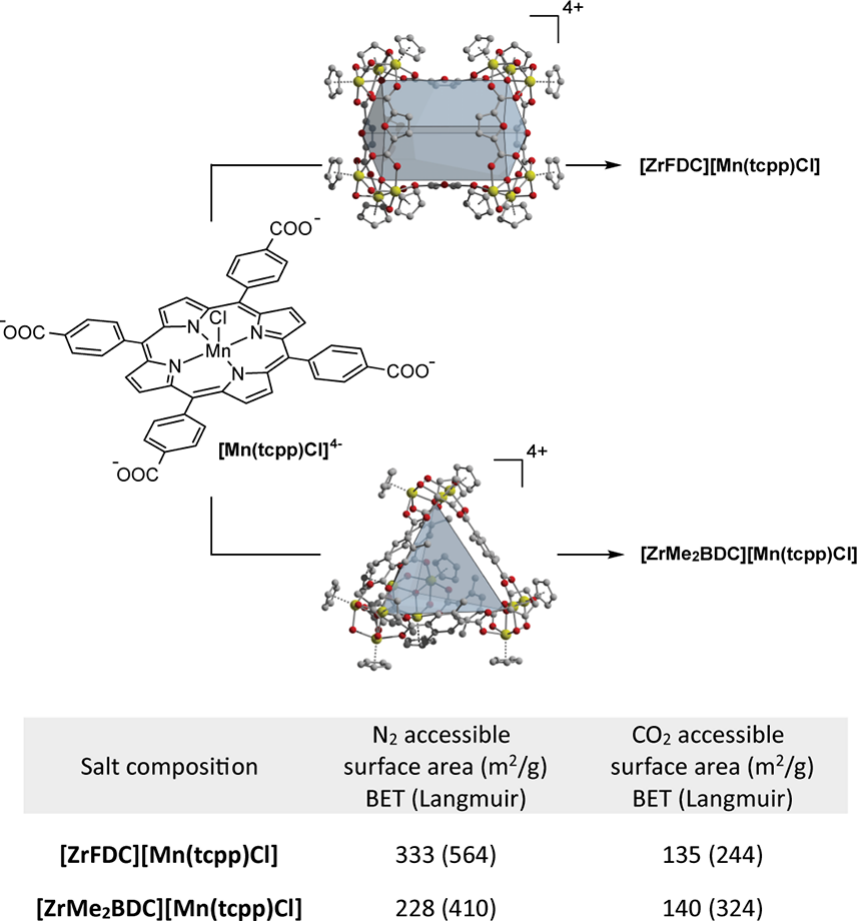
Metathetical synthesis of porous salts. Summary of surface areas determined by N_2_ and CO_2_ adsorption isotherms.

With robust synthetic conditions to prepare metalloporphyrin-based porous salts and inspired by the rich history of layer-by-layer (LbL) assembly of polyelectrolyte thin films,^29^ we targeted the assembly of optically transparent porous thin films comprised of **[Mn(tcpp)Cl]**^**4–**^ and cages **[ZrFDC]**^**4+**^ or **[ZrMe**_**2**_**BDC]**^**4+**^. To this end, we submerged a plasma-treated glass slide in dilute methanolic solutions (0.4 mM) of cationic cages (i.e., **[ZrFDC]OTf**_**4**_ or **[ZrMe**_**2**_**BDC]OTf**_**4**_) followed by a solution of **[HNEt**_**3**_**]**_**4**_**[Mn(tcpp)Cl]**; the growing film was washed with MeOH between each electrolyte dip (Figure 3a). Film deposition and growth were evidenced by visible darkening of the slide upon increasing bilayer deposition cycles. The transmission UV-vis spectrum of the growing film displayed the signals expected for **[Mn(tcpp)Cl]**^**4–**^ incorporation (Figure 3b). Both the intensity of the Soret band (470 nm, Figure S10) and ellipsometry measurements (Figure 3c, Table S1) confirmed a linear relationship between the film thickness and bilayer count. IR and XPS data are consistent with the expected salt formulation and with data obtained for microcrystalline samples of the porous salts (Figures S11 and 12). Once deposited, films are stable to a wide range of solvents (i.e., benzene, chloroform, acetonitrile, THF, ethanol, water, methanol, and DMF) and retain high mechanical stability. Of significance, the film deposition protocol is general; replacement of **[Mn(tcpp)Cl]**^**4–**^ with either unmetalated **H_2_tcpp**^**4–**^ or **[Fe(tcpp)Cl]**^**4–**^ provided optically transparent thin films (Figures S13).

**Figure 3:**
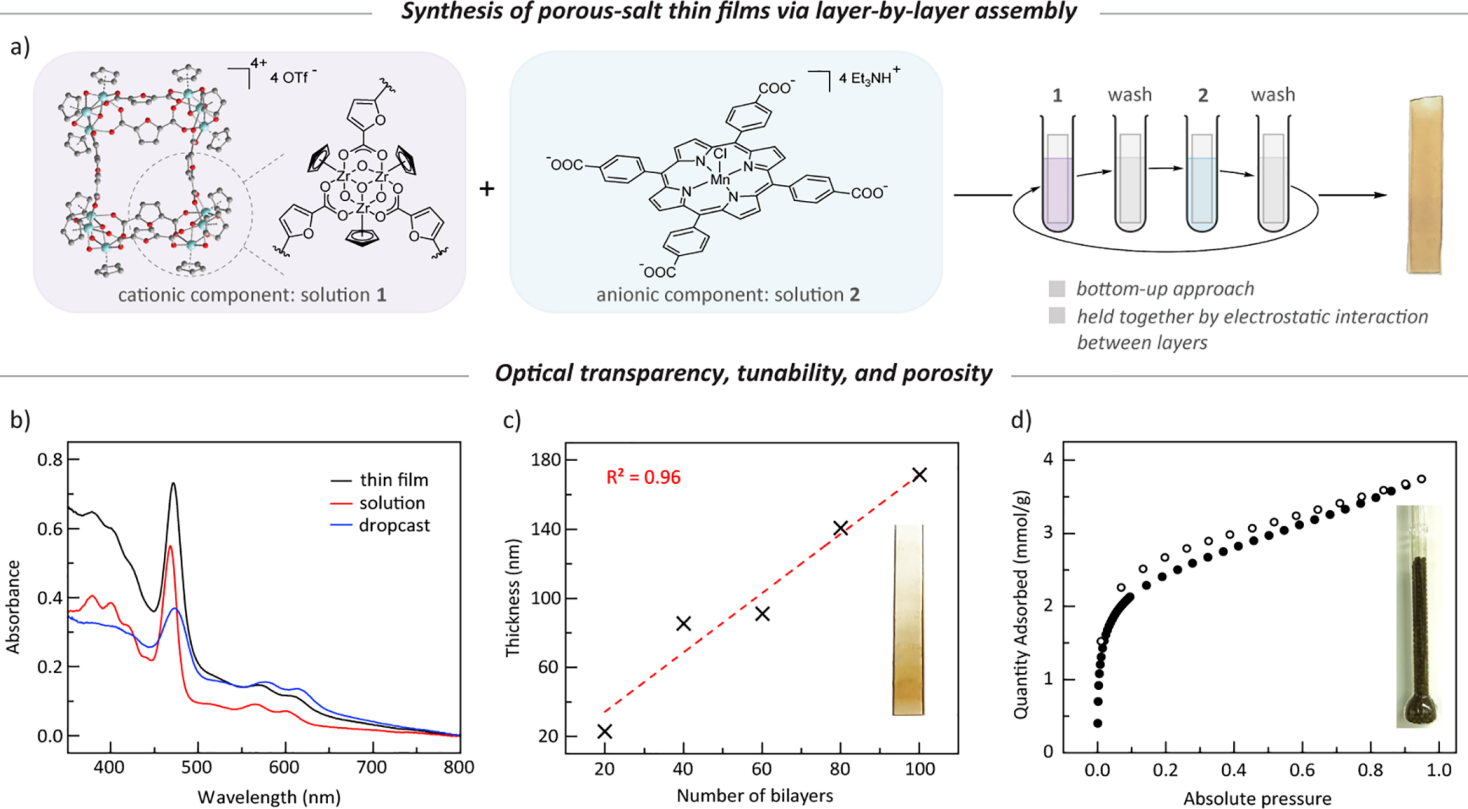
(a) LbL assembly or optically transparent thin films of permanently porous salts. (b) UV-vis spectra of **[HNEt**_**3**_**]**_**4**_**[Mn(tcpp)Cl]** in solution, drop-cast, and a thin film of **[ZrFDC][Mn(tcpp)Cl]**. (c) Ellipsometry measurements indicate a linear relationship between film thickness and bilayer cycles (*R*^2^ = 0.96). (d) CO_2_ adsorption isotherm for a thin film of **[ZrFDC][Mn(tcpp)Cl]** that was activated at 25 °C ((adsorption (●), desorption (○)). The isotherm was measured at 195 K and provided a BET (Langmuir) surface area of 172 (355) m^2^/g.

In order to obtain sufficient quantities of thin films for isothermal gas adsorption studies, we carried out LbL synthesis in glass sample tubes filled with 3 mm diameter glass beads, which were utilized to increase the surface area available for film growth while maintaining sufficient light penetration for subsequent bulk photolysis (Figure 3d). The BET (Langmuir) surface areas of the activated **[ZrFDC][Mn(tcpp)Cl]** and **[ZrMe**_**2**_**BDC][Mn(tcpp)Cl]** films were found to be 172 (355) and 214 (384) m^2^/g, which are in good agreement with the surface areas of bulk powders (i.e., 135 (244) and 140 (324) m^2^/g, respectively).

With access to chemically and mechanically stable porous thin films, we turned our attention to evaluating the potential solid-state photochemistry. Photolysis (λ > 335 nm) of a **[ZrFDC][Mn(tcpp)Cl]** thin film submerged in deoxygenated THF resulted in rapid photoreduction of Mn(III) to Mn(II) evidenced by the complete disappearance of the Mn(III) Soret band (476 nm) and the appearance of the Mn(II) Soret band (446 nm) (Figure 4a). Time-dependent spectra are characterized by negligible background scatter, which enables the visualization of isosbestic points at 418 and 469 nm. This observation demonstrates our low-scatter films to be potential platforms for *in situ* monitoring of solid-state photochemical reactions. The final spectrum overlays well with a spectrum obtained following chemical reduction with NaBH_4_, further supporting the assignment of full Mn(III) to Mn(II) photoreduction (Figure S14). Photolysis in the absence of THF resulted in no photoreduction, which is consistent with THF serving as a halogen-atom trap.^30^ The full conversion of Mn(III) to Mn(II), further corroborated by XPS analysis (Figure S8), indicates that the film is sufficiently porous for THF to diffuse throughout the material. Consistent with the porosity following photoreduction, the BET (Langmuir) surface area as measured by the CO_2_ adsorption isotherm only slightly decreased from 172 (355) to 152 (324) m^2^/g for **[ZrFDC][Mn(tcpp)]** and from 214 (384) to 203 (360) m^2^/g for **[ZrMe**_**2**_**BDC][Mn(tcpp)]**. In contrast, photolysis of microcrystalline samples of porous salts under vacuum or in the presence of solvent did not result in detectable photoreduction as assayed by diffuse reflectance spectroscopy.

**Figure 4:**
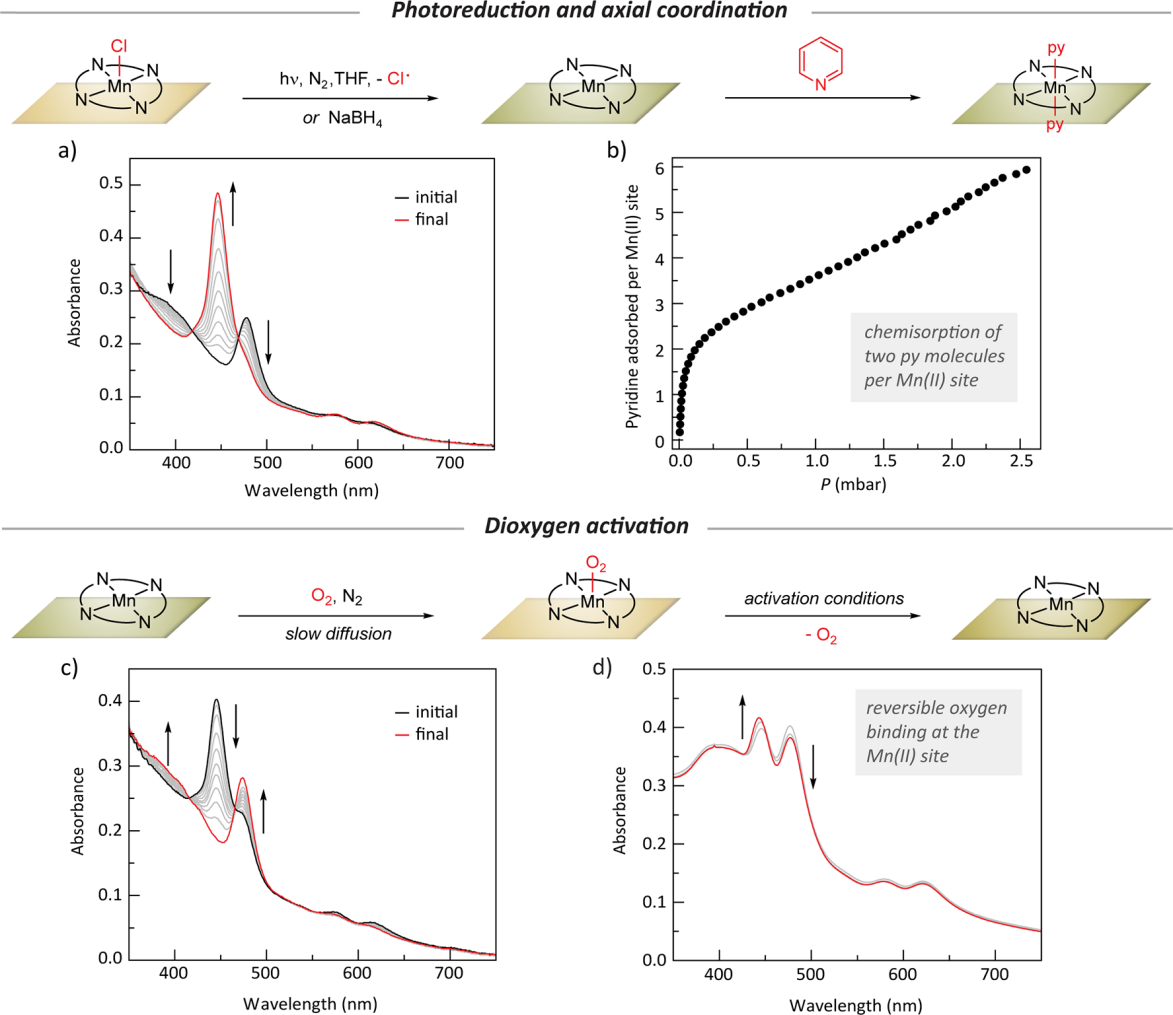
(a) Transmission UV-vis spectra of photoreduction of a **[ZrFDC][Mn(tcpp)Cl]** thin film. (b) Pyridine adsorption isotherm of a **[ZrFDC][Mn(tcpp)]** thin film showing the coordination of two pyridine molecules per Mn(II) site. (c) UV-vis spectra of reaction of a **[ZrFDC][Mn(tcpp)]** thin film with O_2_. (d) UV-vis traces for partial regeneration of **[ZrFDC][Mn(tcpp)]** on activating an oxygenated thin film.

The porosity of the thin films enables the introduction and removal of potential ligands to the confined metal sites. The optical transparency allows those changes to the primary coordination sphere to be observed by *in situ* spectroscopy. As an initial demonstration, we evaluated ligand exchange at the Mn(II) sites of the photoreduced thin films. A methanol solvated **[ZrFDC][Mn(tcpp)]** film displays a Soret band at 442 nm. The addition of pyridine results in an 8 nm shift of the Soret band to 450 nm (Figure S15). A pyridine vapor adsorption isotherm (298 K) confirms that **[ZrFDC][Mn(tcpp)]** displays a sharp uptake of pyridine at low pressure before turning over at ∼0.5 mmol/g (Figure 4b). This value is in good agreement with the expected value of 0.51 mmol/g if each Mn(II) site binds two pyridine ligands.

To further explore site accessibility and small molecule activation at film-confined reactive Mn(II) sites, we exposed a Mn(II)-containing film to O_2_. Over the course of 3 h, *in situ* UV-vis spectroscopy revealed the reappearance of a Mn(III) Soret band which is presumably due to the formation of a Mn(III) superoxide adduct (Figure 4c). O_2_ binding is partially reversible: Warming the oxygenated film to 50 °C under vacuum resulted in the reemergence of the Mn(II) spectral features (Figure 4d). Similarly, reversible O_2_ binding was observed for Mn(II) phthalocyanine complexes, which form Mn(III) superoxide adducts.^31^ Previous studies of O_2_ activation at lattice-confined metalloporphyrins characterized O_2_ binding by single-crystal X-ray diffraction.^32^ While these studies provide structural insight, real time kinetic data are not available because the measurements are intrinsically *ex situ*. The ability to monitor chemical reactions at confined metal sites in real time, both during photoreduction and subsequent chemical reactions, underscores the importance of optically transparent porous films as platforms to study solid-state organometallic chemistry.

In summary, we demonstrate that optically transparent thin films composed of permanently porous molecular cages and photochemically active small molecules can be assembled. LbL assembly allows for precise control over thickness and porosity. The method efficiently site-isolates nonporous molecules within a porous matrix and enables real-time observation of chemical processes at confined metal sites by *in situ* optical spectroscopy. In the films presented here, the Mn sites are chemically accessible as a result of film porosity and are photochemically addressable in the film by virtue of its optical transparency. Specifically, photoreduction, ligand exchange, and O_2_ activation processes are all demonstrated and observed in real time. This new class of materials, which can be synthesized through a straightforward yet tunable method, will significantly advance solid-state photochemistry and provide a platform to study chemical processes in synthetic porous materials by *in operando* spectroscopy. We anticipate that this material platform will play a pivotal role in pushing the boundaries of solid-state photochemistry and facilitate further advancements in heterogeneous catalysis research.

## Supporting Information Available

Experimental methods, synthetic schemes, and spectral, diffraction, thermal, microscopy and gas adsorption data/images (Figures S16–S42) (PDF)
