# Supplementary material for: Unlocking Solid-State Organometallic Photochemistry with Optically Transparent, Porous Salt Thin Films
Source: J Am Chem Soc. 2023 Nov 8;145(46):25068–73. doi: 10.1021/jacs.3c09188 (PMC10863064; doi:10.1021/jacs.3c09188)
Supplement: Supplementary file 1 — ja3c09188_si_001.pdf [file ja3c09188_si_001.pdf]

Supporting Information

Unlocking Solid-State Organometallic Photochemistry with Optically Transparent, Porous Salt Thin Films

Aishanee Sur[†,‡], Joe D. Simmons[∇,‡], Andrew A. Ezazi[†], Kyle J. Korman[∇], Subham Sarkar[†], Ethan T. Iverson[†], Eric D. Bloch[∇,*], David C. Powers[†,*]

[†]*Department of Chemistry, Texas A&M University, College Station, TX 77843, United States*
[∇]*Department of Chemistry, Indiana University, Bloomington, IN 47405, United States*
[‡]*These authors contributed equally to this work.*

Email: edbloch@iu.edu; powers@chem.tamu.edu

Table of Contents

A. General Considerations S3

B. Synthesis and Characterization S5

C. Supporting Data for the Salts S10

D. Thickness Measurements of **[ZrFDC][Mn(tcpp)Cl]** Thin Films S19

E. Supporting Data for Thin Films S22

F. Photolysis Procedures S25

F.1 Photoreduction Procedure for **[ZrFDC][Mn(tcpp)Cl]** Film S25

F.2 Photoreduction Procedure for **[ZrFDC][Mn(tcpp)Cl]** Film on Glass Beads S26

F.3 Photolysis Procedure of Bulk Powder S26

G. Chemical Reduction of Mn(III) on **[ZrFDC][Mn(tcpp)Cl]** Thin Film S27

H. Pyridine Coordination S28

I. O_2 Activation and Reversible Binding S29

I.1 O_2 Activation by the **[ZrFDC][Mn(tcpp)]** Thin Film S29

I.2 Reversibility of O_2 Activation S30

J. Additional Data S31

K. References S57

A. General Considerations

Materials Solvents were obtained as ACS reagent grade. $MnCl_2 \cdot 4H_2O$, pyrrole, 4-formylbenzoic acid, triethylamine, zirconocene dichloride, 2,5-furandicarboxylic acid, 2,5-dimethylbezenedicarboxylic acid, silver triflate, sodium borohydride ($NaBH_4$), methanol (MeOH), chloroform, *N*,*N*-dimethylformamide (DMF), *N*,*N*-dimethylacetamide (DMA), tetrahydrofuran (THF), pyridine, pentane, benzene, acetonitrile, and ethanol (EtOH) were obtained from Fisher Scientific. NMR solvents were purchased from Cambridge Isotope Laboratories. Anhydrous MeOH was obtained by distillation from calcium hydride under N_2 or taken from a Pure Process Technology solvent system and stored in an N_2 glovebox over 4 angstrom sieves. Anhydrous THF was obtained by distillation from sodium with benzophenone indicator under N_2 or taken from a Pure Process Technology solvent system and stored in an N_2 glovebox over 4 Å sieves. 5,10,15,20-tetrakis(4-carboxyphenyl)porphyrin[1] and H_4Mn(tcpp)Cl[2] were prepared according to literature methods or purchased from Combi-Blocks Incorporated.

Characterization Details NMR spectra were recorded on Bruker Avance NEO 400 NMR or Varian 400 MHz Inova NMR operating at 400.09 MHz for ^1H acquisitions and were referenced against solvent signal: CD_3OD (3.28 ppm, ^1H).[3] ^1H NMR data are reported as follows: chemical shift (δ, ppm), multiplicity (s (singlet), d (doublet), t (triplet), m (multiplet), br (broad), integration. TGA measurements were conducted on a TA Instruments TGA 5500. Samples were heated to 600 °C at a rate of 5 °C/min under a 25 mL/min N_2 flow. IR spectra were collected on a Bruker Alpha II FT-IR. XPS measurements were collected on a PHI VersaProbe II Scanning X-ray Microprobe. SEM and EDX were collected with an Auriga 60 FIB-SEM. Film thickness measurements were obtained using an Alpha-SE ellipsometer equipped with 623.8 nm laser. PXRD measurements were carried out on a Philips Xpert Powder X-ray diffractometer (Cu Kα, 1.5418 Å; 40 kV, 25 mA) fitted with a Pixos 2000 detector. The angular range was measured from 5.00 to 45.00° (2θ) with steps of 0.010° and a measurement time of 0.3 second per step.

Gas/Vapor Adsorption and Desorption Details Gas adsorption and desorption isotherms for pressures 0−1.0 bar were measured volumetrically using a Micromeritics 3Flex and Micromertics Tristar II Plus. Samples were transferred under an N_2 atmosphere to analysis tubes which were preweighed under 1 bar of He. The samples were evacuated under high vacuum at 25 °C for at least 24 hours. At this time the tube was weighed under 1 bar of He to determine the mass of the activated sample. The tube was transferred to the analysis port of the 3Flex or Tristar II Plus. UHP-grade (99.999% purity) CO_2 and He were used for all adsorption measurements. For all isotherms, free-space measurements were carried out with He; CO_2 isotherms were measured at −78.15 °C. Acquisition of the pyridine vapor isotherm was carried out as follows: In an N_2 glovebox, a preweighed gas adsorption sample tube was loaded with glass beads coated with 12.0 mg of a thin film of **[ZrFDC][Mn(tcpp)]**. The sample was placed under dynamic vacuum at 25 °C for 24 hours. Anhydrous, airfree pyridine was loaded into the 3Flex vapor dosing tube and freeze-pump-thawed three times

prior to the vapor isotherm. The pyridine vapor isotherm was collected at 25 °C using a thermostated water bath for temperature control.

UV-vis Spectroscopy UV-vis spectra were collected on a Jasco V750 UV-Visible Spectrophotometer. Continuous scan spectra were collected from 300-900 nm at a scan rate for 400 nm/min and a step size of 0.5 nm. Diffuse reflectance spectra were collected with a 60 mm diameter integrating sphere. A background was collected using $BaSO_4$ before measuring the samples. Samples were prepared for diffuse reflectance under an N_2 atmosphere as a 2% by mass slurry in $BaSO_4$ inside a sealed 4 mL quartz cuvette.

B. Synthesis and Characterization

Synthesis of $[HNEt_3]_4[Mn(tcpp)Cl]$

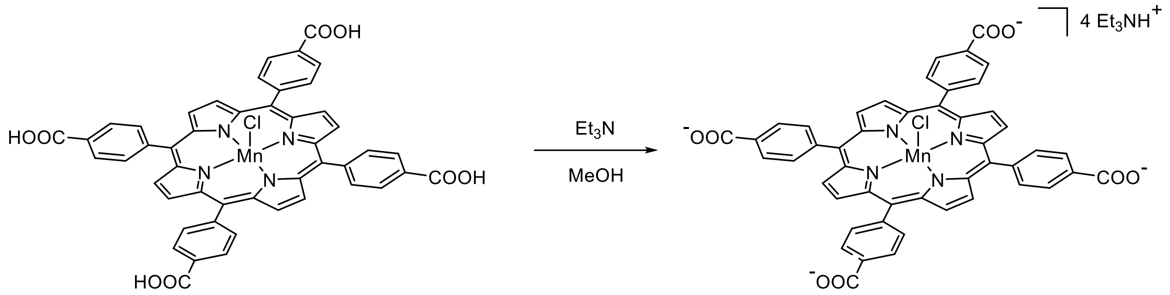

A 20-mL scintillation vial was charged with $H_4Mn(tcpp)Cl$ (0.088 g, 0.10 mmol), methanol (10 mL), and triethylamine (Et_3N) (140 µL, 1.0 mmol). The reaction mixture was stirred at 25 °C for 30 min, at which point all the solids dissolved to make a dark solution. The solution was dried under dynamic vacuum at 45 °C for 1 h to give a dark greenish brown solid of **$[HNEt_3]_4[Mn(tcpp)Cl]$** (0.12 g, 0.093 mmol) in 93% yield. ^1H NMR (δ, 25 °C, 400 MHz, CD_3OD): δ 8.66-6.18 (m, 24H), 3.07 (br, 24H), 1.19 (br, 36H). The obtained UV-vis spectrum is shown in Figure S6 and the obtained IR spectrum is shown in Figure S4.

Synthesis of $Zr_{12}(\mu_3\text{-}O)_4\ (\mu_2\text{-}OH)_{12}(FDC)_6Cl_4$

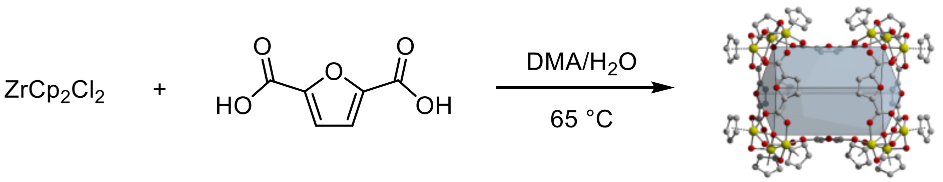

A 20-mL scintillation vial was charged with zirconocene dichloride (0.29 g, 1.0 mmol), 2,5-furandicarboxylic acid (0.078 g, 0.50 mmol), DMA (17 mL) and deionized H_2O (0.50 mL). The reaction mixture was heated to 65 °C for 18 h. The obtained white crystalline solid was collected by centrifugation and the mother liquor was decanted. Activated material was prepared by washing the white solid with fresh DMA 5 times, and then chloroform 5 times, replacing the solvent every 12 h. Chloroform solvated material was then activated at 25 °C under dynamic vacuum to yield the title compound (0.18 g, 0.058 mmol) in 70% yield. The obtained IR spectrum is shown in Figure S4.

Synthesis of $Zr_{12}(\mu_3\text{-}O)_4(\mu_2\text{-}OH)_{12}(FDC)_6(OTf)_4$

$$Zr_{12}(\mu_3\text{-}O)_4(\mu_2\text{-}OH)_{12}(fdc)_6Cl_4 \ + \ AgOTf \xrightarrow[25\ °C]{MeOH} Zr_{12}(\mu_3\text{-}O)_4(\mu_2\text{-}OH)_{12}(fdc)_6(OTf)_4$$

A foil-wrapped 1-L round-bottomed flask was charged with $Zr_{12}(\mu_3\text{-}O)_4(\mu_2\text{-}OH)_{12}(FDC)_6Cl_4$ (2.0 g, 0.62 mmol), silver triflate (0.64 g, 2.5 mmol), and methanol (500 mL). The reaction mixture was stirred in darkness for 3 days. The resulting slurry was centrifuged, and the mother liquor collected. The methanol was removed under reduced pressure resulting in a white solid. The methanol solvated material was activated at 25 °C under dynamic vacuum to yield the title compound (1.8 g, 0.50 mmol) in 80% yield. The obtained UV-vis spectrum is shown in Figure S6.

Synthesis of [ZrFDC][Mn(tcpp)Cl] salt

$$Zr_{12}(\mu_3\text{-}O)_4(\mu_2\text{-}OH)_{12}(fdc)_6(OTf)_4 \ + \ [HNEt_3]_4[Mn(tcpp)Cl] \xrightarrow[25\ °C]{MeOH} [Zr_{12}(\mu_3\text{-}O)_4(\mu_2\text{-}OH)_{12}(fdc)_6][Mn(tcpp)Cl]$$

A 20-mL scintillation vial was charged with **$[HNEt_3]_4[Mn(tcpp)Cl]$** (0.050 g, 0.038 mmol) and methanol (10 mL). A 100-mL round-bottomed flask was charged with $Zr_{12}(\mu_3\text{-}O)_4(\mu_2\text{-}OH)_{12}(FDC)_6(OTf)_4$ (0.20 g, 0.054 mmol) and methanol (40 mL). The porphyrin solution was added to the round-bottomed flask containing the cage solution. Upon mixing, a brown solid was obtained. The brown solid was washed with methanol using a Soxhlet extractor for 48 h. The methanol solvated material was activated at 25 °C under dynamic vacuum to afford the title compound in nearly quantitative yield.[a] The obtained diffuse reflectance UV-vis spectrum is shown in Figure S6 and the obtained IR spectrum is shown in Figure S4.

Synthesis of $Zr_{12}(\mu_3\text{-}O)_4(\mu_2\text{-}OH)_{12}(Me_2BDC)_6Cl_4$

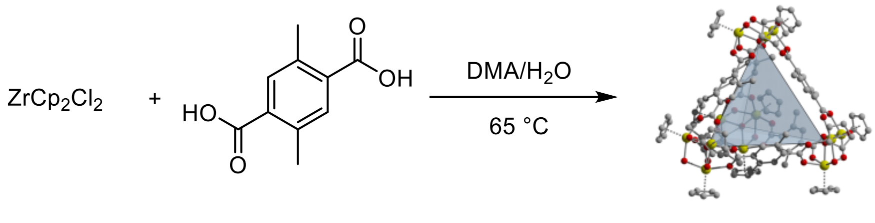

A 20-mL scintillation vial was charged with zirconocene dichloride (0.29 g, 1.0 mmol), 2,5-dimethylbezenedicarboxylic acid (0.097 g, 0.50 mmol), DMA (17 mL), and deionized H_2O (0.50 mL). The reaction mixture was heated to 65 °C for 18 h. The obtained white crystalline solid was collected by centrifugation and the mother liquor was decanted. Activated material

[a] The apparent yield of this reaction was variable and dependent on the level of solvation of specific samples.

was prepared by washing the white solid with fresh DMA 5 times, and then with chloroform 5 times, replacing the solvent every 12 h. Chloroform solvated material was then activated at 25 °C to afford the title compound (0.17 g, 0.049 mmol) in 60% yield. The obtained IR spectrum is shown in Figure S5.

Synthesis of $Zr_{12}(\mu_3\text{-}O)_4(\mu_2\text{-}OH)_{12}(Me_2BDC)_6(OTf)_4$

$$Zr_{12}(\mu_3\text{-}O)_4(\mu_2\text{-}OH)_{12}(Me_2bdc)_6Cl_4 + AgOTf \xrightarrow[25\ °C]{MeOH} Zr_{12}(\mu_3\text{-}O)_4(\mu_2\text{-}OH)_{12}(Me_2bdc)_6(OTf)_4$$

A foil-wrapped 1-L round-bottomed flask was charged with $Zr_{12}(\mu_3\text{-}O)_4(\mu_2\text{-}OH)_{12}(Me_2BDC)_6Cl_4$ (2.0 g, 0.58 mmol), silver triflate (0.59 g, 2.3 mmol), and methanol (500 mL). The reaction was stirred in darkness for 3 days. The resulting slurry was centrifuged, and the mother liquor was collected. The methanol was removed under reduced pressure resulting in a white solid. The methanol solvated material was activated at 25 °C under dynamic vacuum to afford the title compound (1.7 g, 0.46 mmol) in 79% yield. The obtained UV-vis spectrum is shown in Figure S7.

Synthesis of $[ZrMe_2BDC][Mn(tcpp)Cl]$

$$Zr_{12}(\mu_3\text{-}O)_4(\mu_2\text{-}OH)_{12}(Me_2bdc)_6(OTf)_4 + [HNEt_3]_4[Mn(tcpp)Cl] \xrightarrow[25\ °C]{MeOH} [Zr_{12}(\mu_3\text{-}O)_4(\mu_2\text{-}OH)_{12}(Me_2bdc)_6][Mn(tcpp)Cl]$$

A 20-mL scintillation vial was charged with **$[HNEt_3]_4[Mn(tcpp)Cl]$** (0.050 g, 0.038 mmol) and methanol (10 mL). A 100-mL round-bottomed flask was charged with $Zr_{12}(\mu_3\text{-}O)_4(\mu_2\text{-}OH)_{12}(Me_2BDC)_6(OTf)_4$ (0.20 g, 0.052 mmol) and methanol (40 mL). The porphyrin solution was added to the round-bottomed flask containing the cage solution. Upon mixing, a brown solid was obtained. The resulting brown solid wash washed with methanol using a Soxhlet extractor for 48 h. The methanol solvated material was activated at 25 °C under dynamic vacuum to afford the title compound in nearly quantitative yield.[b] The obtained diffuse reflectance UV-vis spectrum is shown in Figure S7 and the obtained IR spectrum is shown in Figure S5.

[b] The apparent yield of this reaction was variable and dependent on the level of solvation of specific samples.

Preparation of a thin film of [ZrFDC][Mn(tcpp)Cl] on a glass slide

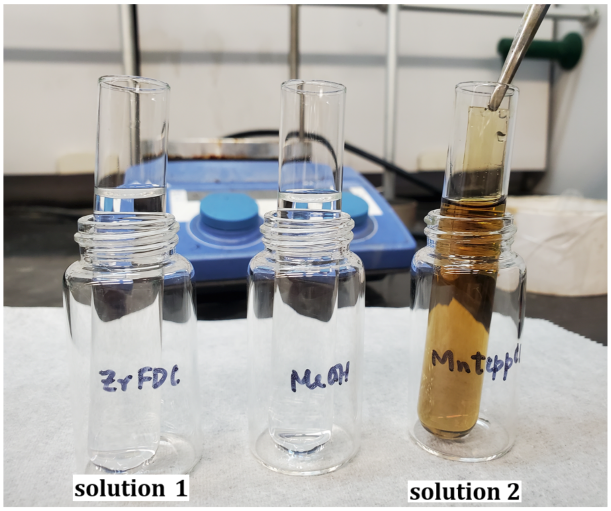

Solution 1 was prepared by charging a test tube with $Zr_{12}(\mu_3\text{-}O)_4(\mu_2\text{-}OH)_{12}(FDC)_6(OTf)_4$ (0.015 g, 0.0041 mmol) and methanol (10 mL). **Solution 2** was prepared by charging a test tube with **$[HNEt_3]_4$[Mn(tcpp)Cl]** (0.0050 g, 0.0040 mmol) and methanol (10 mL). A plasma-treated glass slide (42 mm × 9 mm) was dipped in **solution 1** for 5 min. The slide was dipped in fresh MeOH for 10 s. The washed slide was then dipped in **solution 2** for 15 s. The slide was dipped in MeOH for 10 s. Subsequent bilayer depositions were made by sequential dips in **solution 1** (15 s), MeOH (10 s), **solution 2** (15 s), MeOH (10 s). After each 10 bilayers, the wash solutions were replaced with fresh MeOH. The bilayer formation process was repeated until the desired thickness was achieved.

Preparation of thin films of [ZrFDC][Mn(tcpp)Cl] on glass beads

Solution 1 was prepared by charging a 100-mL Schlenk flask with **$[HNEt_3]_4$[Mn(tcpp)Cl]** (0.10 g, 0.076 mmol) and MeOH (50 mL). **Solution 2** was prepared by charging a separate 100-mL Schlenk flask with $Zr_{12}(\mu_3\text{-}O)_4(\mu_2\text{-}OH)_{12}(FDC)_6(OTf)_4$ (0.10 g, 0.028 mmol) and MeOH (50 mL). **Solution 1** was transferred via cannula into a gas adsorption sample tube filled with 3 mm glass beads and then removed by the same cannula. The sample tube and glass beads were then washed with MeOH via cannula transfer and removal. **Solution 2** was transferred via cannula into the gas adsorption tube and was removed by the same cannula. The sample tube and glass beads were then washed with MeOH via cannula transfer and removal. This sequence constitutes a single bilayer. This process was repeated 200 times, replacing the cage and porphyrin solutions every 25 bilayers. The resulting film was additionally washed with MeOH 5 more times. The methanol solvated material was activated at 25 °C under dynamic vacuum to yield 0.0016 g of brownish green film on glass beads.

Preparation of thin films of [ZrMe_2BDC][Mn(tcpp)Cl] on glass beads

Solution 1 was prepared by charging a 100-mL Schlenk flask with **[$HNEt_3$]$_4$[Mn(tcpp)Cl]** (0.10 g, 0.076 mmol) and MeOH (50 mL). **Solution 2** was prepared by charging a separate 100-mL Schlenk flask with $Zr_{12}(\mu_3\text{-}O)_4(\mu_2\text{-}OH)_{12}(Me_2BDC)_6(OTf)_4$ (0.10 g, 0.026 mmol) and MeOH (50 mL). **Solution 1** was transferred via cannula into a gas adsorption sample tube filled with 3 mm glass beads and then removed by the same cannula. The sample tube and glass beads were then washed with MeOH via cannula transfer and removal. **Solution 2** was transferred via cannula into the gas adsorption tube and was removed by the same cannula. The sample tube and glass beads were then washed with MeOH via cannula transfer and removal. This sequence constitutes a single bilayer. This process was repeated 200 times, replacing the cage and porphyrin solutions every 25 bilayers. The resulting film was additionally washed with MeOH 5 more times. The methanol solvated material was activated at 25 °C under dynamic vacuum to yield 0.00032 g of brownish green film on glass beads.

C. Supporting Data for the Salts

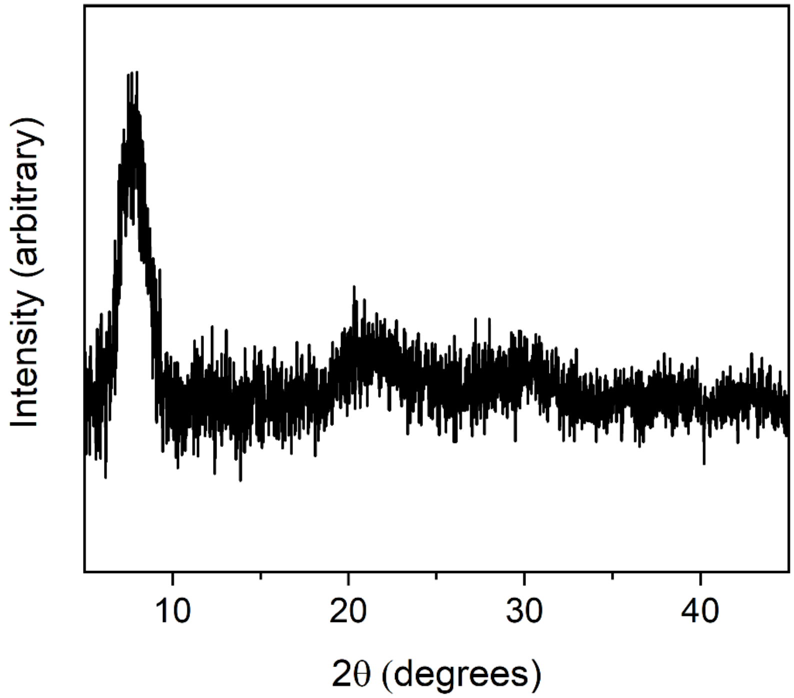

Figure S1. Powder X-ray diffraction pattern of **[ZrFDC][Mn(tcpp)Cl]**.

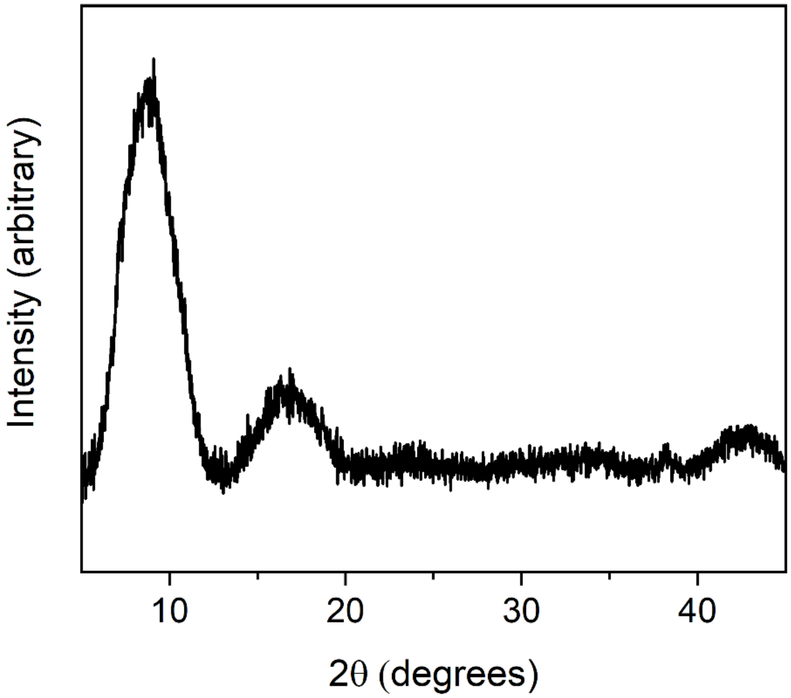

Figure S2. Powder X-ray diffraction pattern of **[ZrMe$_2$BDC][Mn(tcpp)Cl]**.

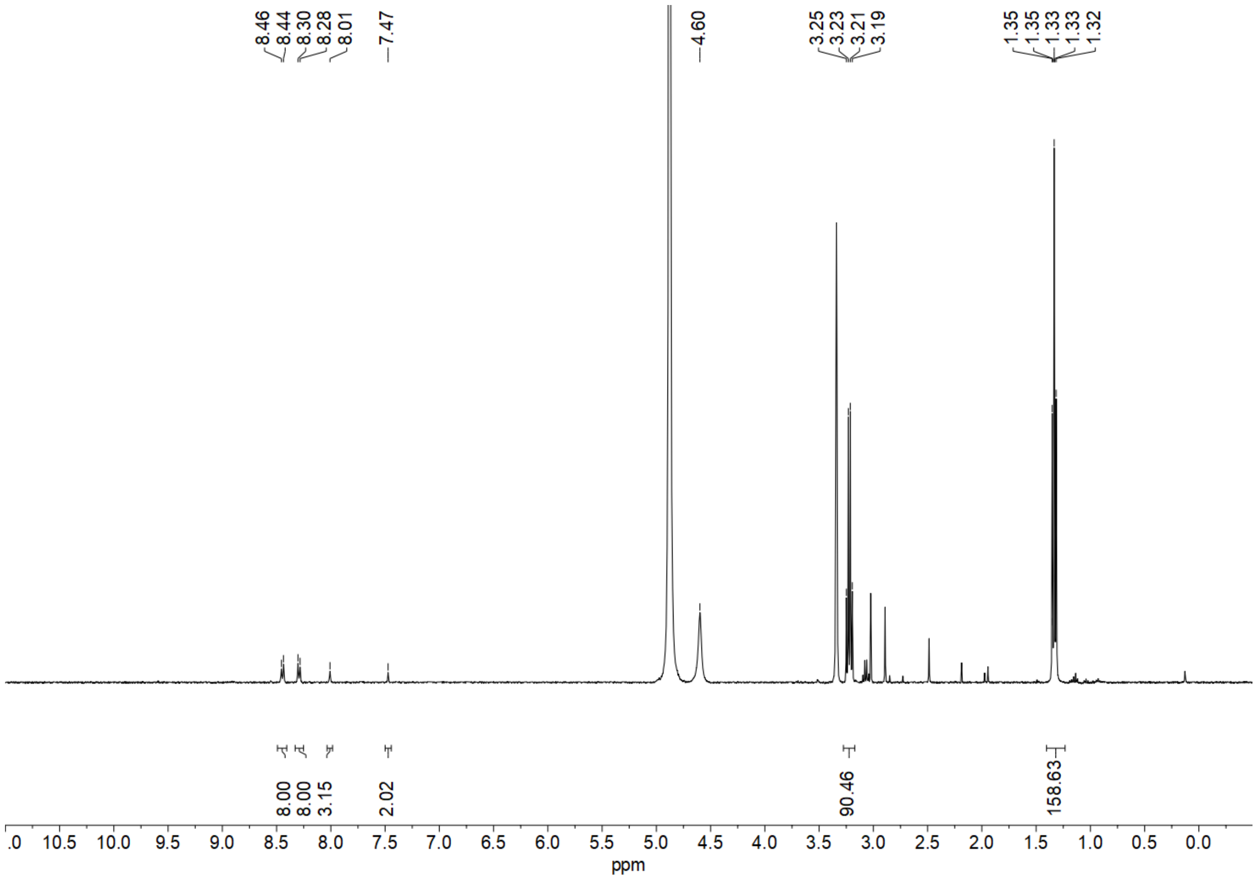

Figure S3. ^{1}H NMR spectrum of a supernatant solution resulting from a salt metathesis of **[ZrFDC][H_2(tcpp)]** in CD_3OD (400 MHz) at 25 °C.

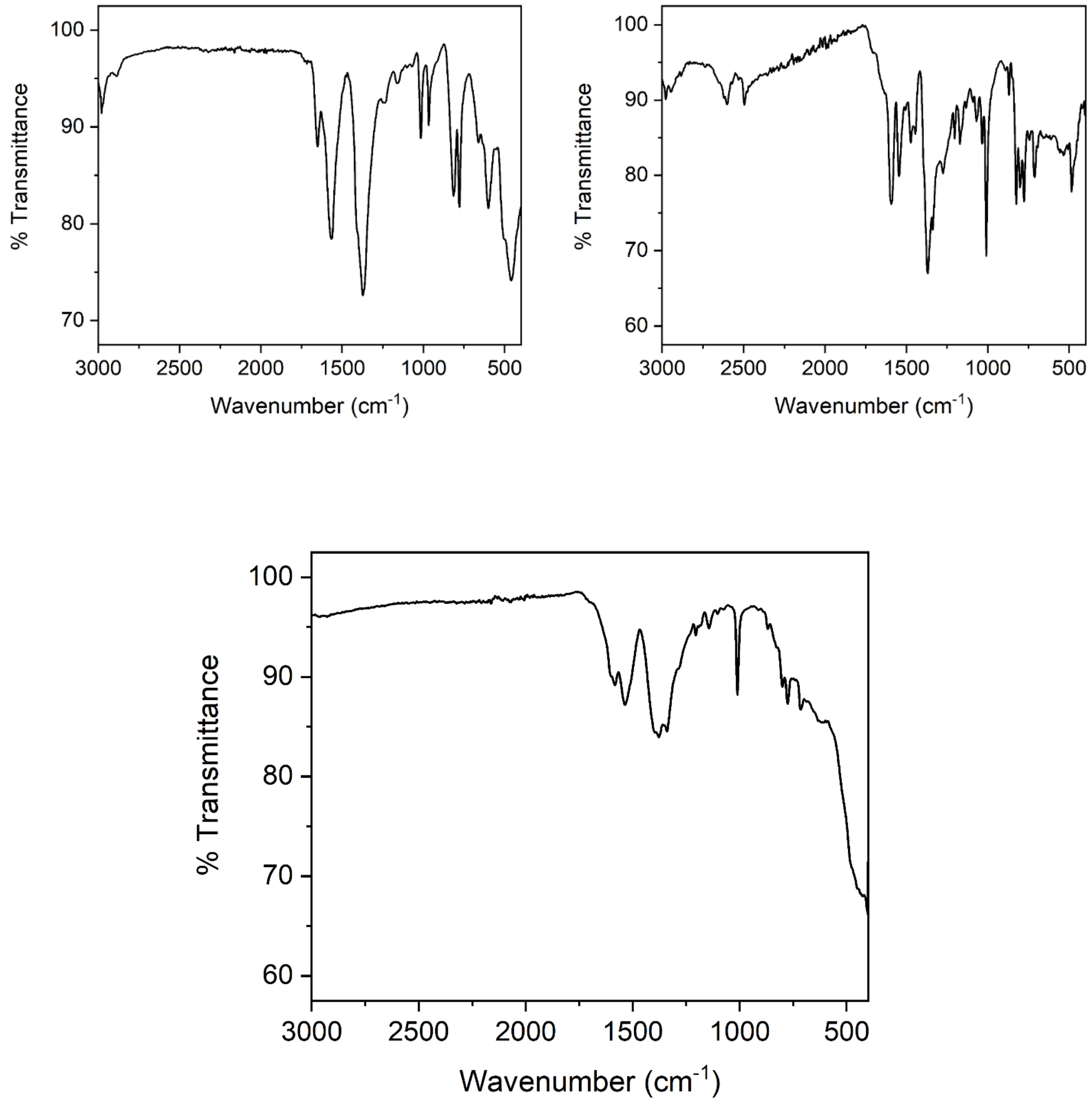

Figure S4. IR spectra of activated $Zr_{12}(\mu_3\text{-}O)_4(\mu_2\text{-}OH)_{12}(FDC)_6Cl_4$ (top left), **$[HNEt_3]_4$[Mn(tcpp)Cl]** (top right) and activated **[ZrFDC][Mn(tcpp)Cl]** (bottom).

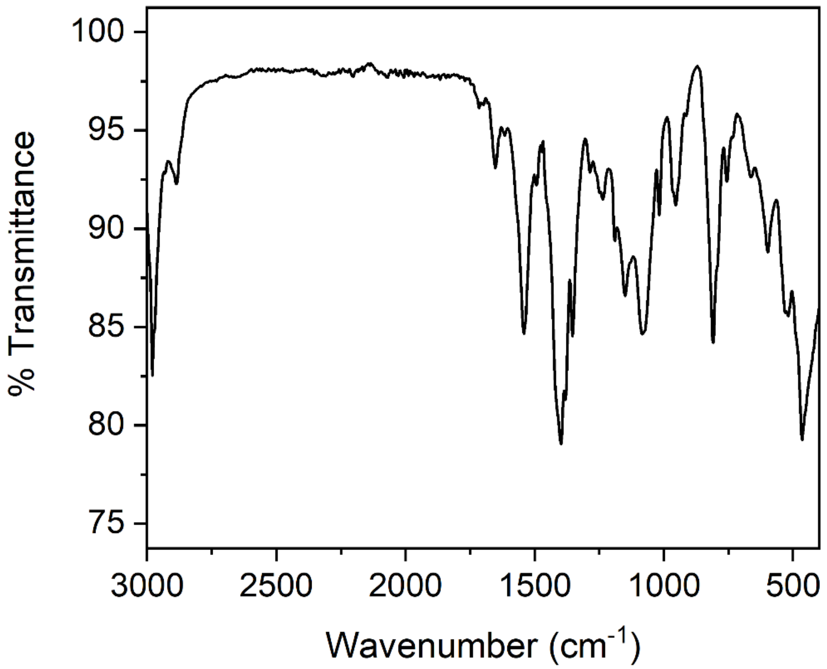

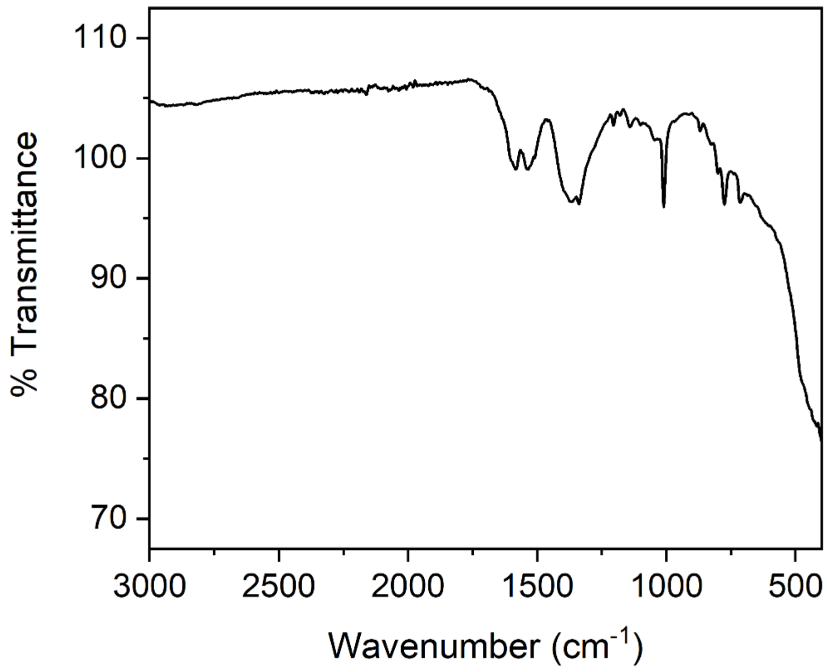

Figure S5. IR spectra of activated $Zr_{12}(\mu_3\text{-}O)_4(\mu_2\text{-}OH)_{12}(Me_2BDC)_6Cl_4$ (top) and activated **[ZrMe$_2$BDC][Mn(tcpp)Cl]** (bottom).

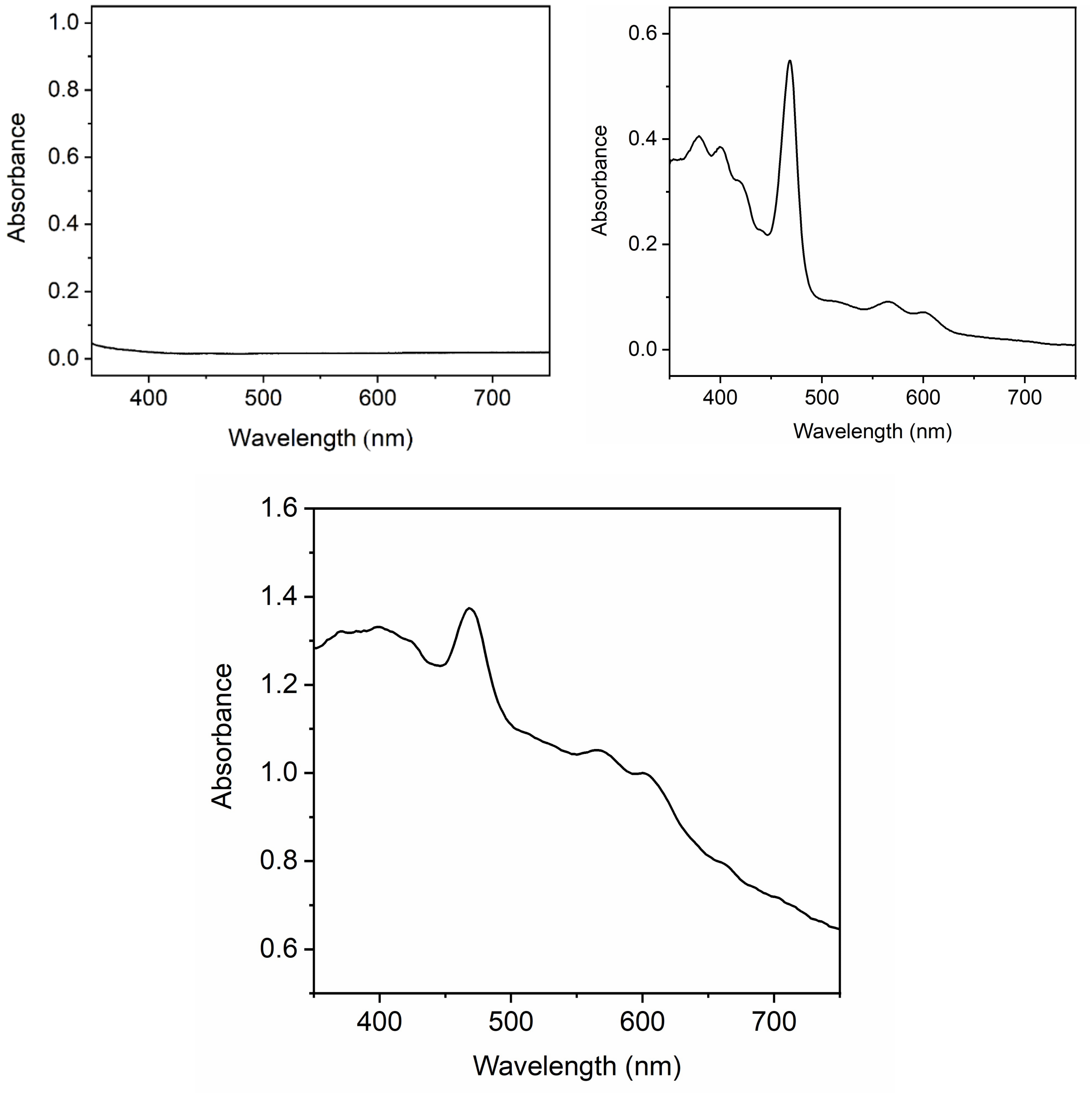

Figure S6. Transmission UV-vis spectrum of $Zr_{12}(\mu_3\text{-}O)_4(\mu_2\text{-}OH)_{12}(FDC)_6(OTf)_4$ (0.1 M) in methanol (top left) and **$[HNEt_3]_4$[Mn(tcpp)Cl]** (15 μM) in methanol (top right). Diffuse reflectance UV-vis spectrum of **[ZrFDC][Mn(tcpp)Cl]** powder (bottom). The spectrum obtained following photolysis of **[ZrFDC][Mn(tcpp)Cl]** powder in THF does not show any change.

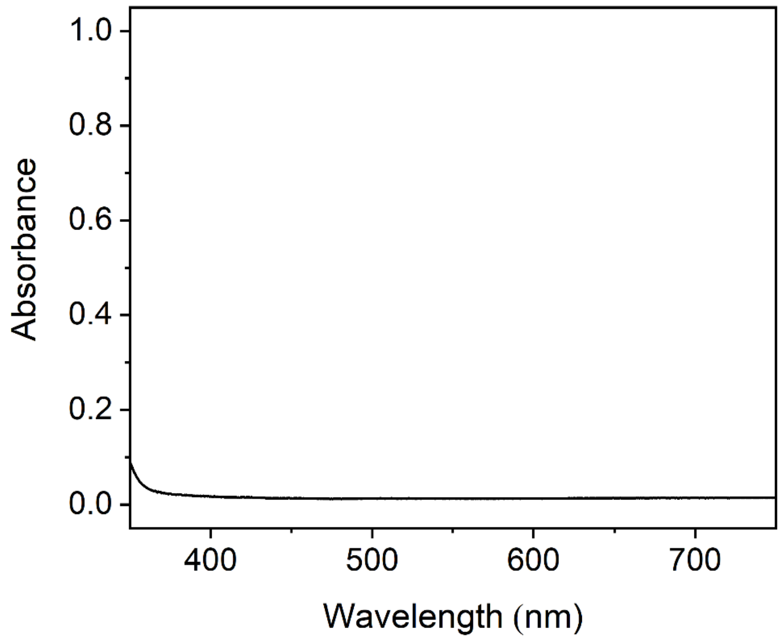

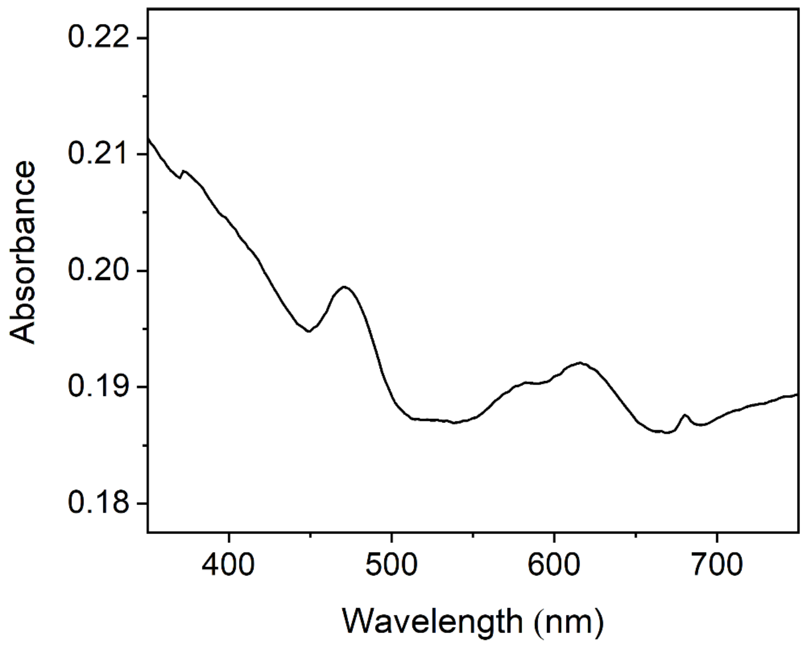

Figure S7. Transmission UV-vis spectrum of $Zr_{12}(\mu_3\text{-}O)_4(\mu_2\text{-}OH)_{12}(Me_2BDC)_6(OTf)_4$ (0.1 M) in methanol (top) and diffuse reflectance UV-vis spectrum of **[ZrMe$_2$BDC][Mn(tcpp)Cl]** powder (bottom). The spectrum obtained following photolysis of **[ZrMe$_2$BDC][Mn(tcpp)Cl]** powder in THF does not show any change.

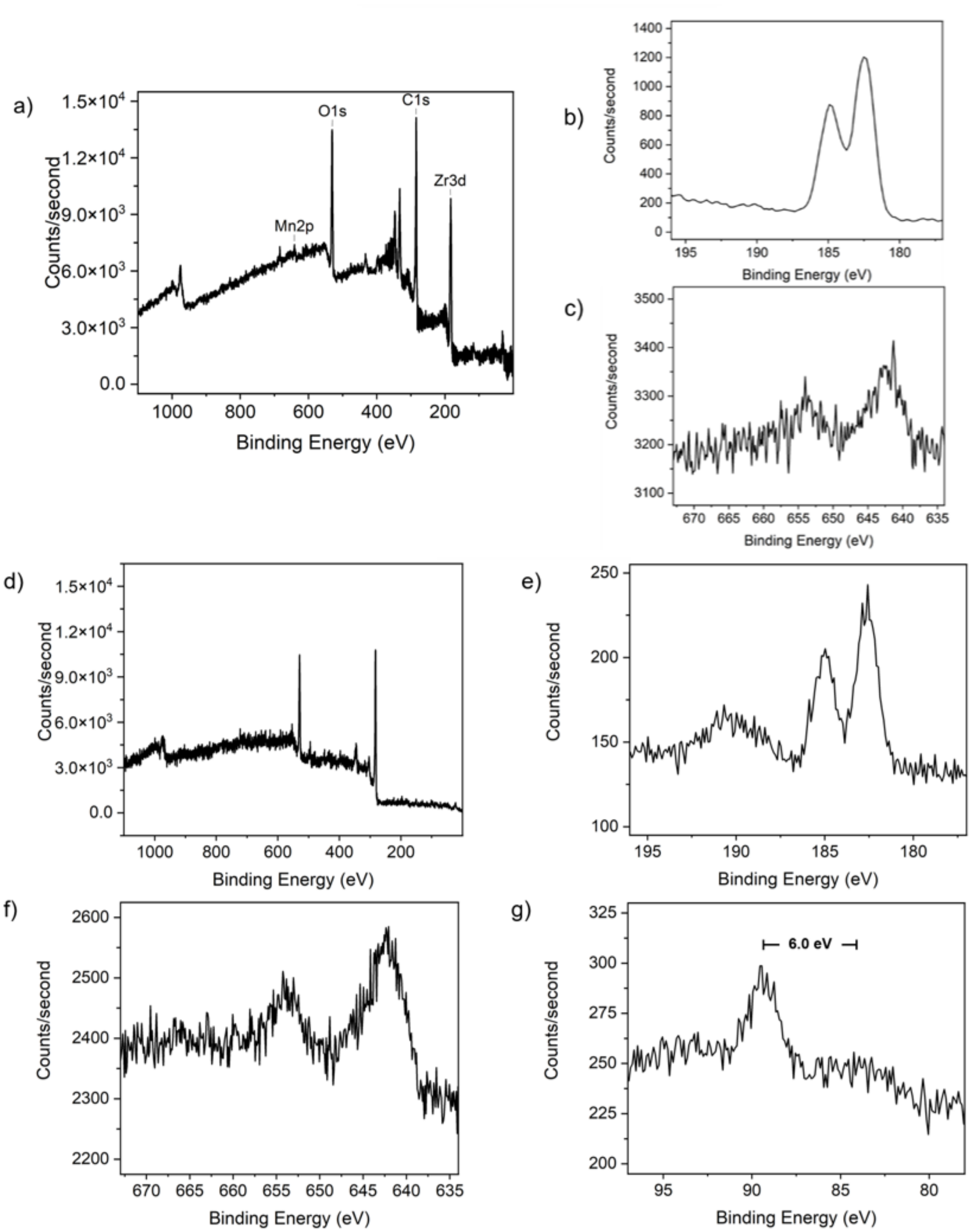

Figure S8. X-ray photoelectron spectroscopy a) survey, b) high resolution Zr3d, and c) high resolution Mn2p spectra of **[ZrFDC][Mn(tcpp)Cl]**, d) Survey of **[ZrFDC][Mn(tcpp)]** film e) Zr3d of film f) Mn2p of film g) Mn3s of film with a 6.0 eV peak split indicative of Mn(II) oxidation state.

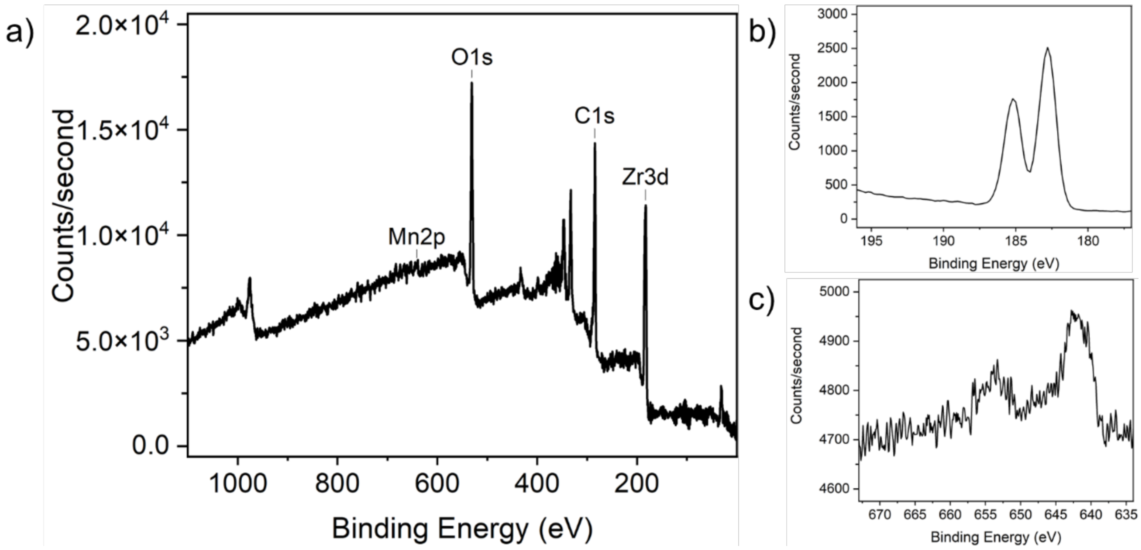

Figure S9. X-ray photoelectron spectroscopy a) survey, b) high resolution Zr3d, and c) high resolution Mn2p spectra of **[ZrMe$_2$BDC][Mn(tcpp)Cl]**.

D. Thickness Measurements of [ZrFDC][Mn(tcpp)Cl] Thin Films

D.1 UV-vis absorption as a function of thickness

Solution 1 was prepared by charging a test tube with $Zr_{12}(\mu_3\text{-}O)_4(\mu_2\text{-}OH)_{12}(FDC)_6(OTf)_4$ (0.015 g, 0.0041 mmol) and methanol (10 mL).It was diluted by adding 1 mL of **solution 1** to 9 mL of MeOH to give **solution 1a**. **Solution 2** was prepared by charging a test tube with **$[HNEt_3]_4[Mn(tcpp)Cl]$** (0.0050 g, 0.0040 mmol) and methanol (10 mL). It was diluted by adding 1 mL of **solution 2** to 9 mL of MeOH to give **solution 2a**. A plasma-treated glass slide (42 mm x 9 mm) was dipped in **solution 1a** for 5 min. The slide was dipped in fresh MeOH for 10 s. The washed slide was then dipped in **solution 2a** for 15 s. The slide was dipped in MeOH for 10 s. Subsequent bilayer depositions were made by sequential dips in **solution 1a** (15 s), MeOH (10 s), **solution 2a** (15 s), MeOH (10 s). After each 10 bilayers the UV-vis absorbance of the film was recorded while sitting in MeOH inside the cuvette.

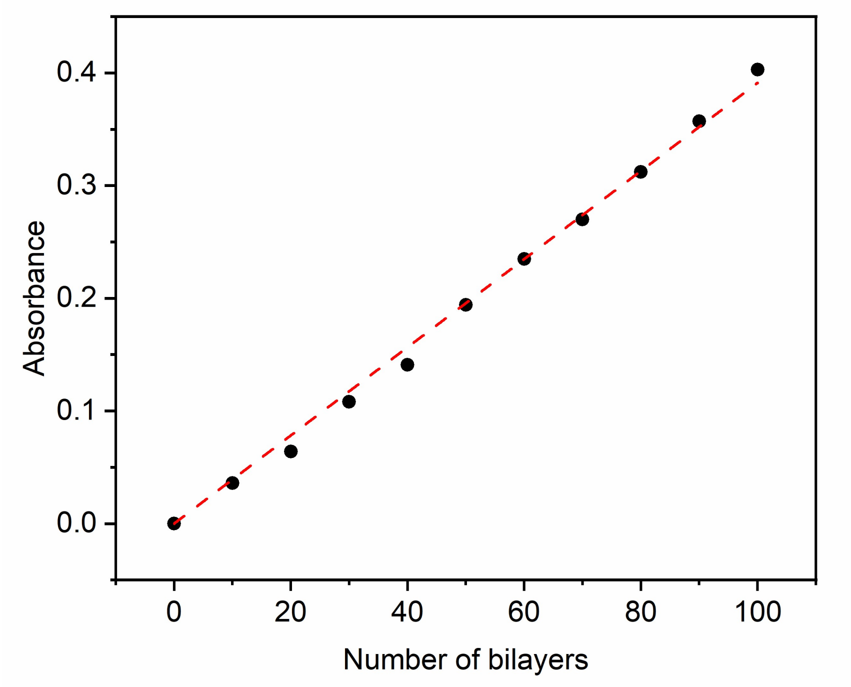

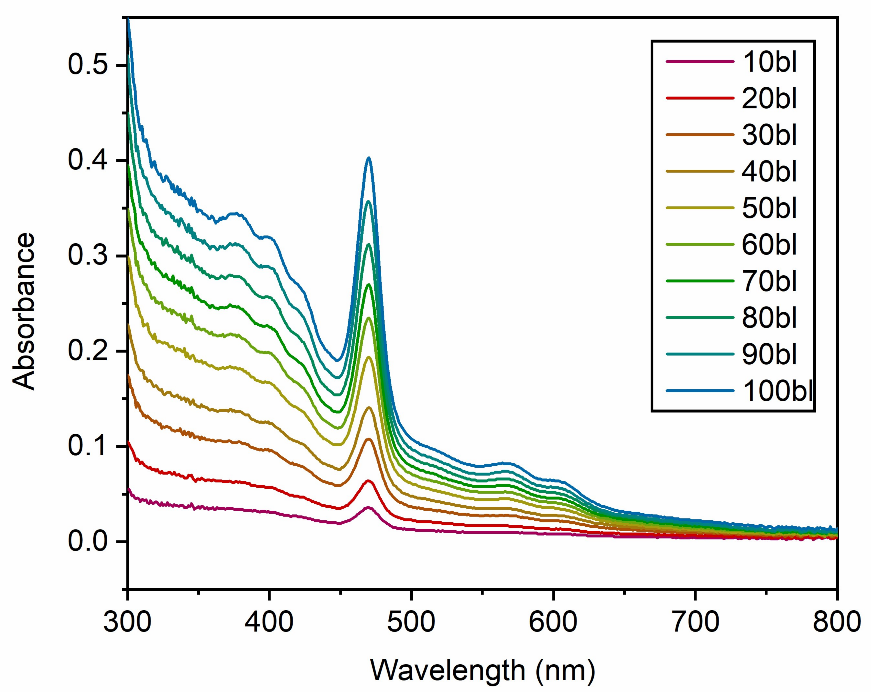

Figure S10. Plot of absorbance of the thin films of **[ZrFDC][Mn(tcpp)Cl]** at 470 nm vs. number of bilayers (top). Transmission UV-vis spectrum of thin-films of **[ZrFDC][Mn(tcpp)Cl]** of different bilayers in methanol (bottom).

D.2 Ellipsometry of a striped film

Solution 1 was prepared by charging a test tube with $Zr_{12}(\mu_3\text{-}O)_4(\mu_2\text{-}OH)_{12}(FDC)_6(OTf)_4$ (0.015 g, 0.0041 mmol) and methanol (10 mL). **Solution 2** was prepared by charging a test tube with **$[HNEt_3]_4[Mn(tcpp)Cl]$** (0.0050 g, 0.0040 mmol) and methanol (10 mL). 60 mm (from bottom) of a plasma-treated glass slide (80 mm × 9 mm) was dipped in **solution 1** for 30 s. The slide was dipped in fresh MeOH for 30 s. The washed slide was then dipped in **solution 2** for 30 s. The slide was dipped in MeOH for 30 s. Subsequent bilayer depositions were made by sequential dips in **solution 1** (30 s), MeOH (30 s), **solution 2** (30 s), MeOH (30 s). This process was repeated 20 times to form 20 bilayers. Then 48 mm (from bottom) of the slide was treated similarly to deposit the next 20 bilayers. The dipping height of the slide was consecutively decreased by 12 mm after deposition of each 20 bilayers. The number of bilayers varied on the film from 20 to 100 with an interval of 20 to give a striped film with 5 distinct regions. Thickness of each region was measured by ellipsometry and the thickness value was recorded as an average of 5 measurements at different spots on a particular region.

Table S1. Ellipsometry data of the striped film

Number of bilayers	Average thickness (nm)	Standard error (nm)
20	23	13
40	85	3.1
60	90	2.6
80	140	0.24
100	171	2.6

E. Supporting Data for Thin Films

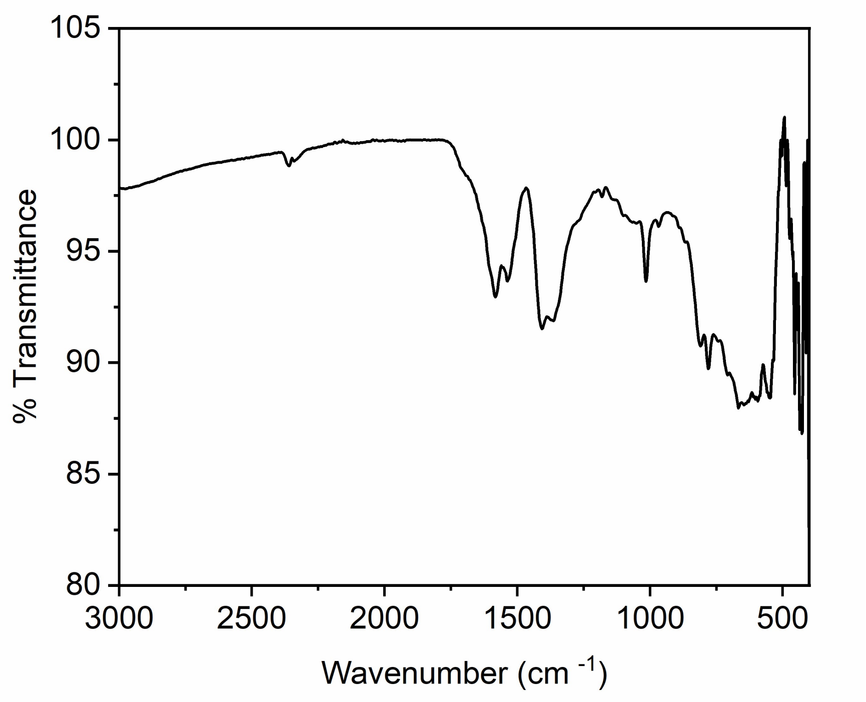

Figure S11. IR spectrum of **[ZrFDC][Mn(tcpp)Cl]** film scraped from a glass slide.

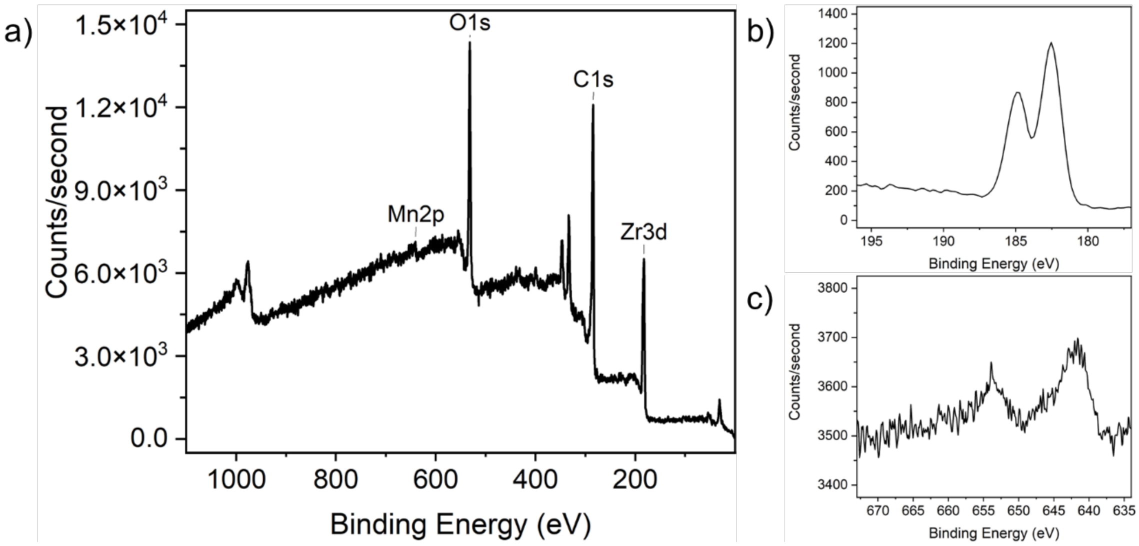

Figure S12. X-ray photoelectron spectroscopy a) survey, b) high resolution Zr3d, and c) high resolution Mn2p spectra of **[ZrFDC][Mn(tcpp)Cl]** film.

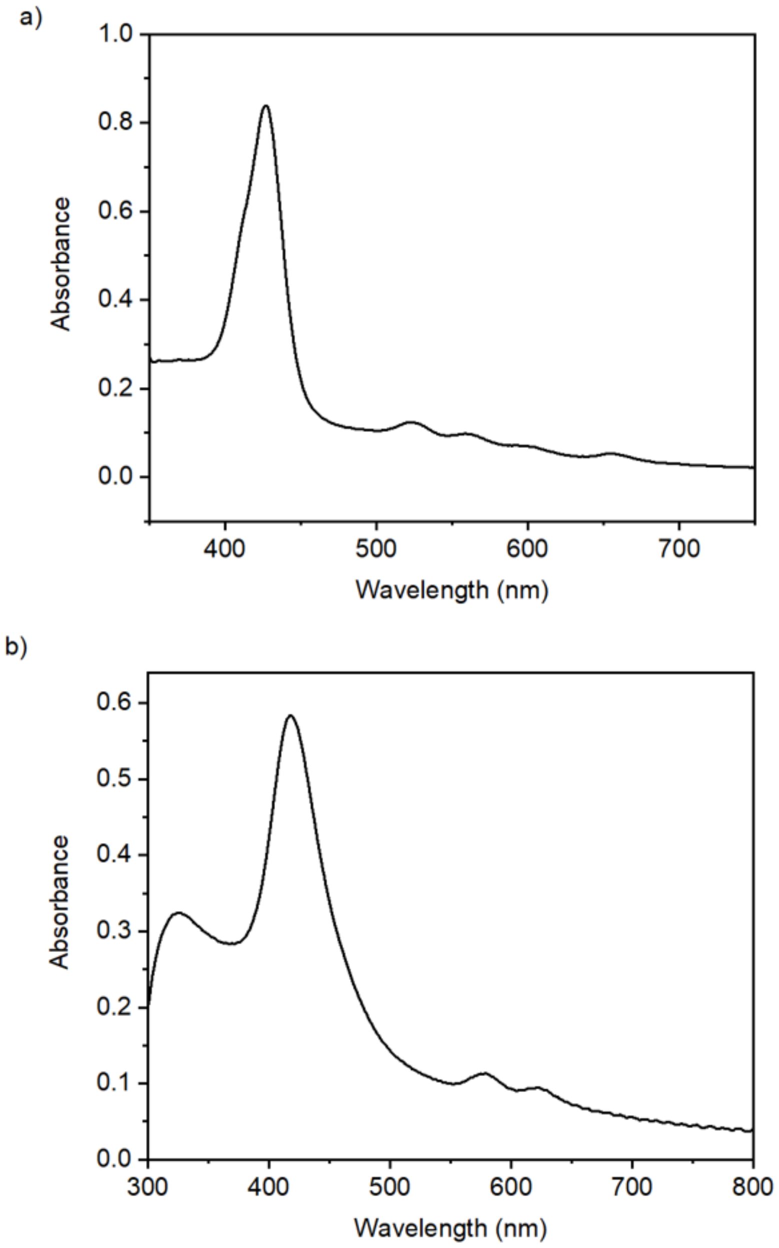

Figure S13. Transmission UV-vis spectra of a) **[ZrFDC][tcpp]** film and b) **[ZrFDC][Fe(tcpp)Cl]** film.

F. Photolysis Procedures

F.1 Photoreduction Procedure for [ZrFDC][Mn(tcpp)Cl] Film

A 70-bilayer film was placed in a quartz cuvette with an attached stopcock (pictured below) and activated under a dynamic vacuum at 25 °C for 16 h. After activation, anhydrous THF (3.5 mL) was added to the cuvette under a nitrogen atmosphere and the cuvette was stoppered. The film was then photolyzed with Hg-vapor light source fitted with a 335 nm longpass filter for 6 h. The conversion of Mn(III) to Mn(II) was monitored by UV-vis spectroscopy, showing the disappearance of the Soret band at 476 nm and the appearance of that at 446 nm.

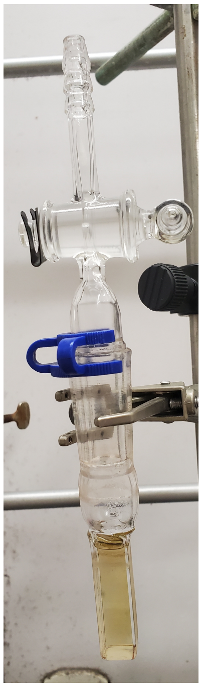

F.2 Photoreduction Procedure for [ZrFDC][Mn(tcpp)Cl] Film on Glass Beads

A 200-bilayer film was grown in a gas adsorption sample tube filled with 3 mm glass beads and was activated under dynamic vacuum at 25 °C for 16 h. After activation, anhydrous THF (10 mL) was added to the gas adsorption tube. The film was then photolyzed with a broad band Hg-vapor lamp for 12 h. The conversion of Mn(III) to Mn(II) was presumed complete by the observed color change from brownish green to bright green matching that seen in previous photolysis experiments.

F.3 Photolysis Procedure of Bulk Powder

A gas adsorption sample tube was charged with activated **[ZrFDC][Mn(tcpp)Cl]** salt powder (21.1 mg) and anhydrous THF (10 mL) was added to the sample tube under a N_2 atmosphere. The salt was photolyzed under a broad band Hg-vapor light source for 12 hours. Diffuse reflectance UV-vis spectra of before and after photolysis showed no evidence of Mn(III) to Mn(II) photoreduction.

G. Chemical Reduction of Mn(III) on [ZrFDC][Mn(tcpp)Cl] Thin Film

A 200-bilayer film was placed in a quartz cuvette with an attached stopcock and activated under a dynamic vacuum at 25 °C for 16 h. After activation, anhydrous MeOH (3.5 mL) was added to the cuvette under a nitrogen atmosphere. $NaBH_4$ (0.00038 g, 0.10 mmol) was added and the cuvette was stoppered. The conversion of Mn(III) to Mn(II) was monitored by UV-vis spectroscopy, showing the disappearance of the Soret band at 472 nm and the appearance of that at 442 nm. Reduction was complete in 15 minutes. The noise in the baseline generated due to evolution of H_2 bubbles. If the film was kept in the solution for longer time, leaching due to reaction with excess $NaBH_4$ was observed.

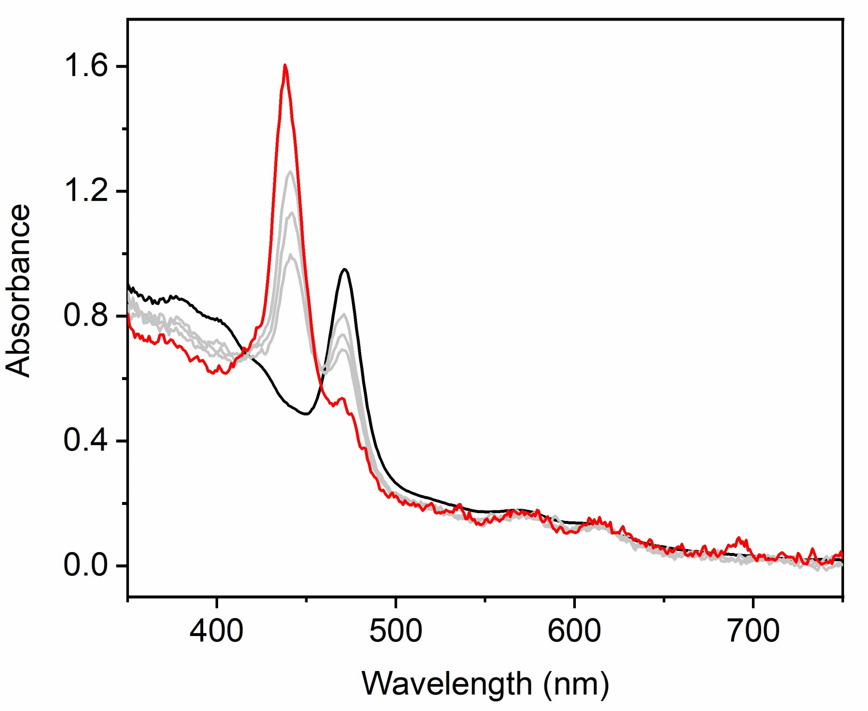

Figure S14. Transmission UV-vis spectrum of a thin film of **[ZrFDC][Mn(tcpp)Cl]** in MeOH (—) and that of **[ZrFDC][Mn(tcpp)]** after treating with $NaBH_4$ in MeOH (—).

H. Pyridine Coordination

A 100-bilayer film of **[ZrFDC][Mn(tcpp)]** was photoreduced by the method described in Section F.1, but instead of THF, the reduction was done in MeOH. The Soret peak of Mn(II) in MeOH has a λ_{max} at 442 nm. After complete reduction, under a nitrogen atmosphere, the MeOH was decanted, the cuvette was refilled with pentane, and 300 μL of distilled pyridine was added. The absorption spectrum recorded after 5 minutes showed a redshift of the soret peak to 450 nm. The two Q-bands also showed redshifts (572 nm → 580 nm, and 613 nm → 620 nm). Pyridine coordination also decreased the intensity of the Soret band slightly.

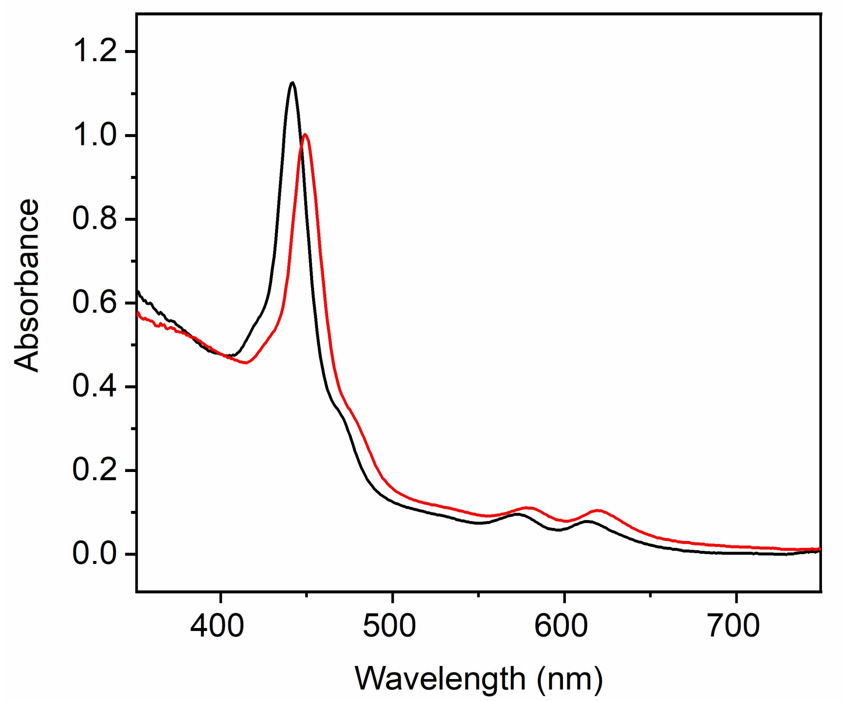

Figure S15. Transmission UV-vis spectrum of a photoreduced thin film of **[ZrFDC][Mn(tcpp)]** in MeOH (—), and after addition of pyridine (—).

I. O_2 Activation and Reversible Binding

I.1 O_2 Activation by the [ZrFDC][Mn(tcpp)] Thin Film

A 70-bilayer film was photoreduced according to the method described in Section B. Under a nitrogen atmosphere, the THF was decanted, and the cuvette was refilled with fresh anhydrous THF. The photoreduced film was soaked in this fresh THF for 16 h to remove any HCl generated during the photoreduction process. The THF wash was decanted and the film was activated under a dynamic vacuum at 25 °C for 16 h. The cuvette was backfilled with N_2, and the stopcock was closed. An O_2-filled balloon was attached to the cuvette and the stopcock was opened to allow slow diffusion of O_2. The conversion of Mn(II) to Mn(III) was monitored by UV-vis spectroscopy, showing the disappearance of the Soret band at 446 nm and the appearance of that at 476 nm.

Exposure of an activated photoreduced film to air instead of an O_2 balloon resulted in rapid oxidation (*i.e.*, complete within 5 minutes) as evidenced by UV-vis spectroscopy.

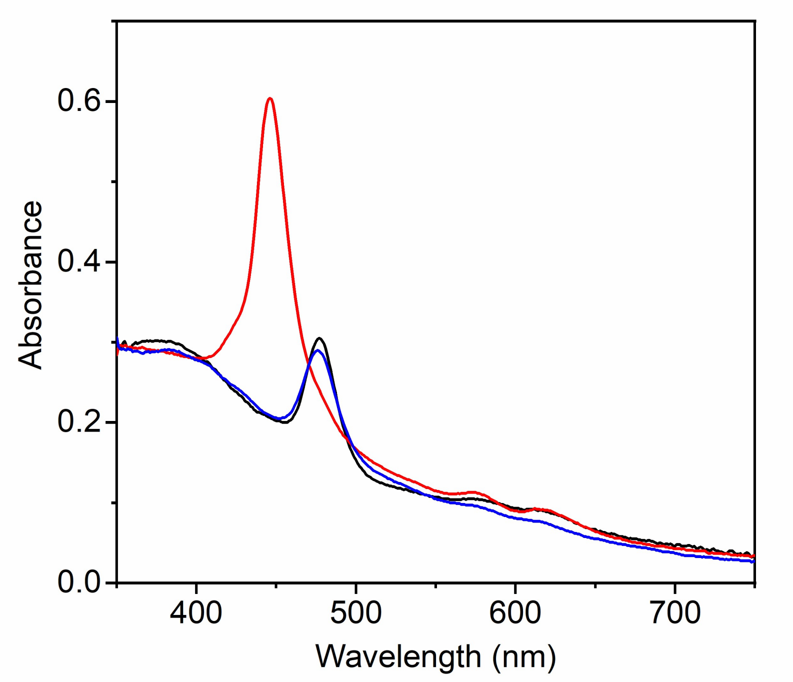

Figure S16. Transmission UV-vis spectrum of **[ZrFDC][Mn(tcpp)Cl]** thin film in THF (—), **[ZrFDC][Mn(tcpp)]** film after photolysis in THF (—), and **[ZrFDC][Mn(tcpp)O_2]** film after exposing the activated photoreduced film to air (—).

I.2 Reversibility of O_2 Activation

A 50-bilayer **[ZrFDC][Mn(tcpp)Cl]** film was grown on the inside of a quartz cuvette. The film was photoreduced in THF using the method reported in Section F.1. The reduced film was activated under dynamic vacuum at 25 °C for 16 h and then placed under 1.000 bar of O_2 and UV-vis spectra were collected periodically. After 3.5 h, the sample was placed under dynamic vacuum for 24 h after which time a UV vis spectrum was collected. The sample was then placed under dynamic vacuum and heated to 100 °C for over 48 h and a UV-vis spectrum was collected. The strong initial Soret band of Mn(II) was seen to decrease in intensity slowly over 3.5 h with a concomitant growth of the Mn(III) band, indicating an oxidation process occurring at the Mn center. Upon subjection to vacuum and subsequent heating, the process was reversed with a decrease in intensity of the Mn(III) Soret band and a regrowth of the Mn(II) band. Spectra are collected in Figure 4 of the main text.

J. Additional Data

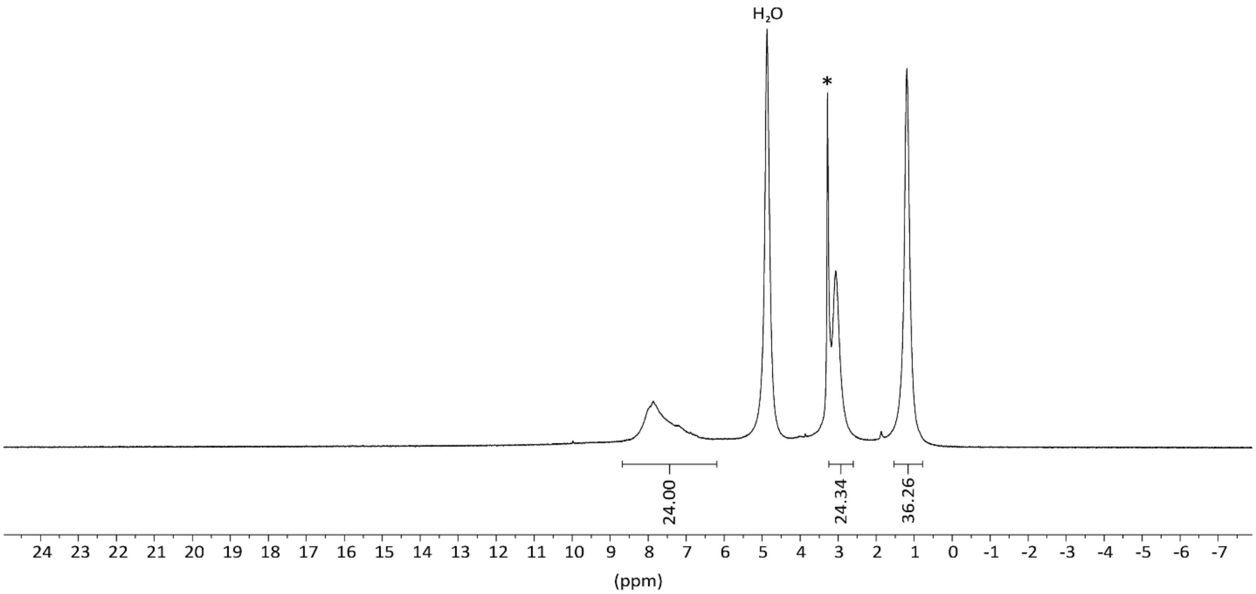

Figure S17. ^{1}H NMR spectrum of **[HNEt$_3$]$_4$[Mn(tcpp)Cl]** in CD_3OD (400 MHz) at 25 °C. CD_3OD solvent peak is marked with *.

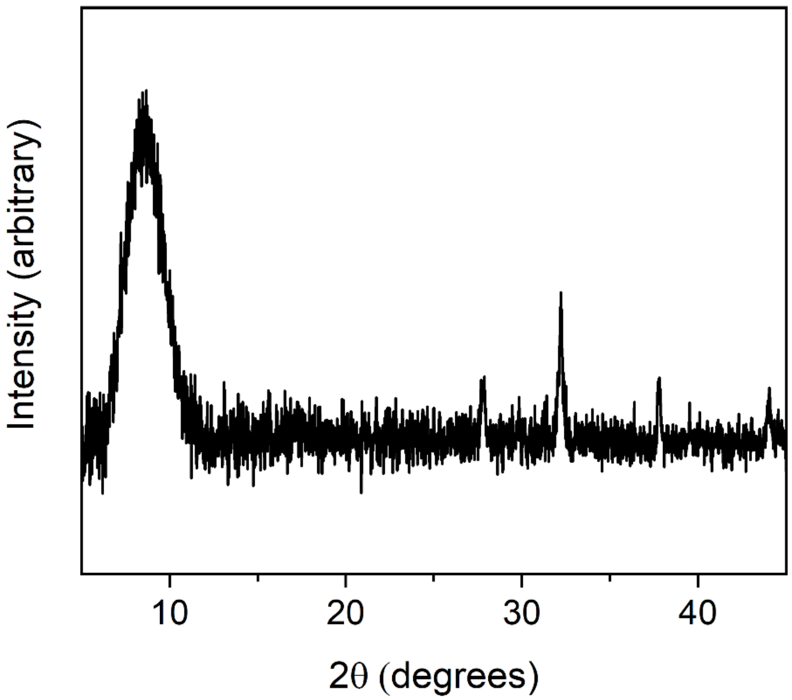

Figure S18. Powder X-ray diffraction pattern of $Zr_{12}(\mu_3\text{-}O)_4(\mu_2\text{-}OH)_{12}(FDC)_6Cl_4$.

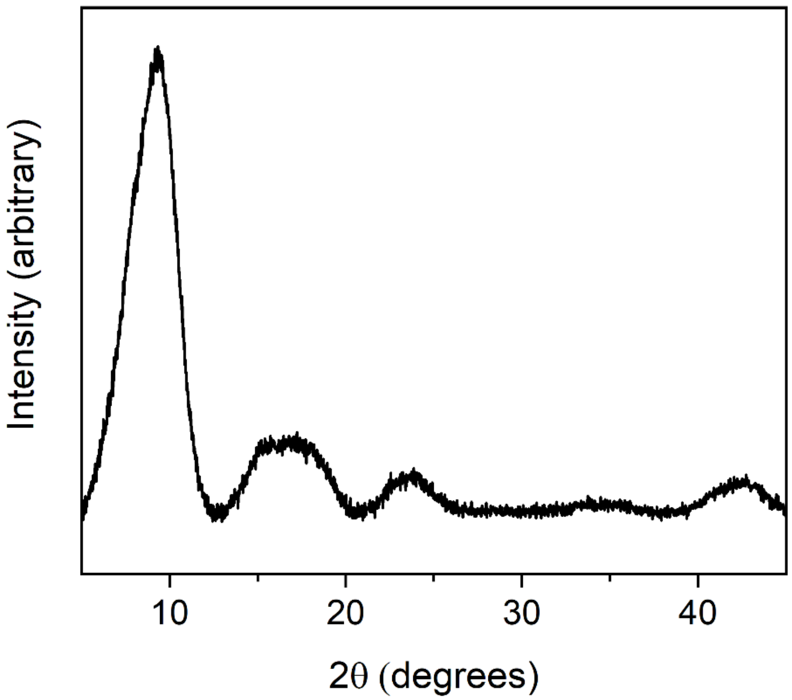

Figure S19. Powder X-ray diffraction pattern of $Zr_{12}(\mu_3\text{-}O)_4(\mu_2\text{-}OH)_{12}(Me_2BDC)_6Cl_4$.

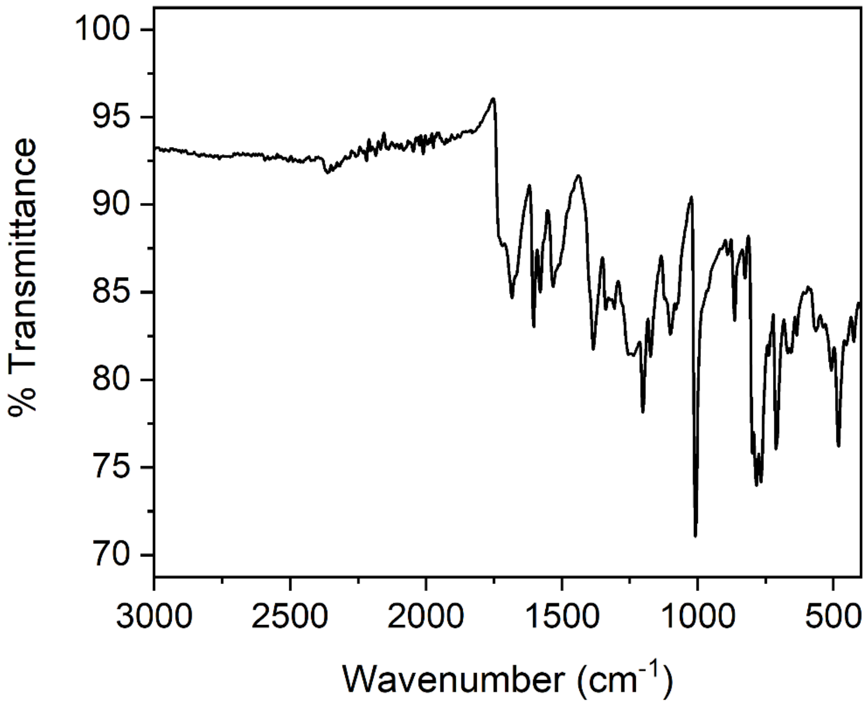

Figure S20. IR spectrum of $H_4Mn(tcpp)Cl$.

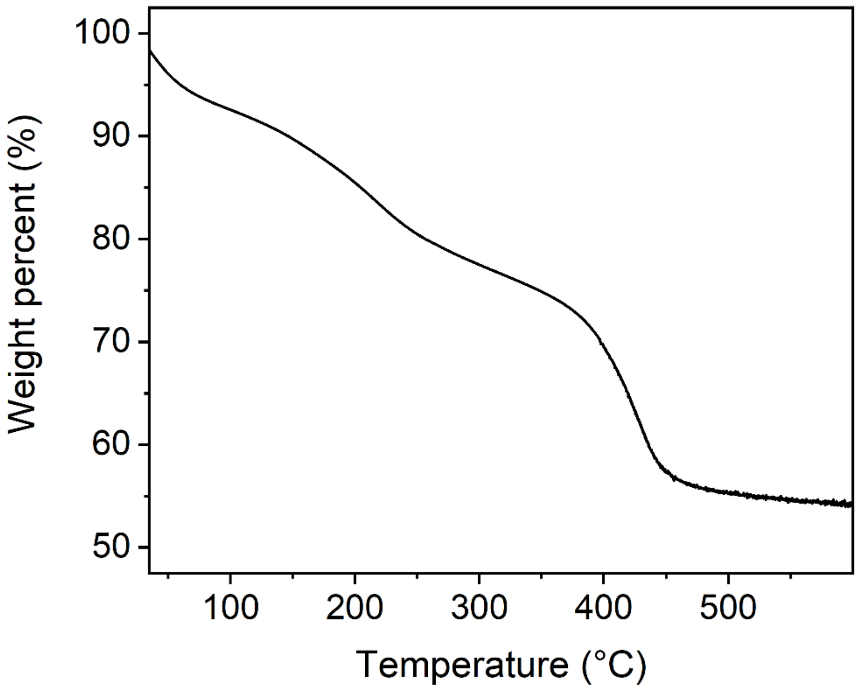

Figure S21. Thermal gravimetric analysis of $CHCl_3$-washed $Zr_{12}(\mu_3\text{-}O)_4(\mu_2\text{-}OH)_{12}(FDC)_6Cl_4$.

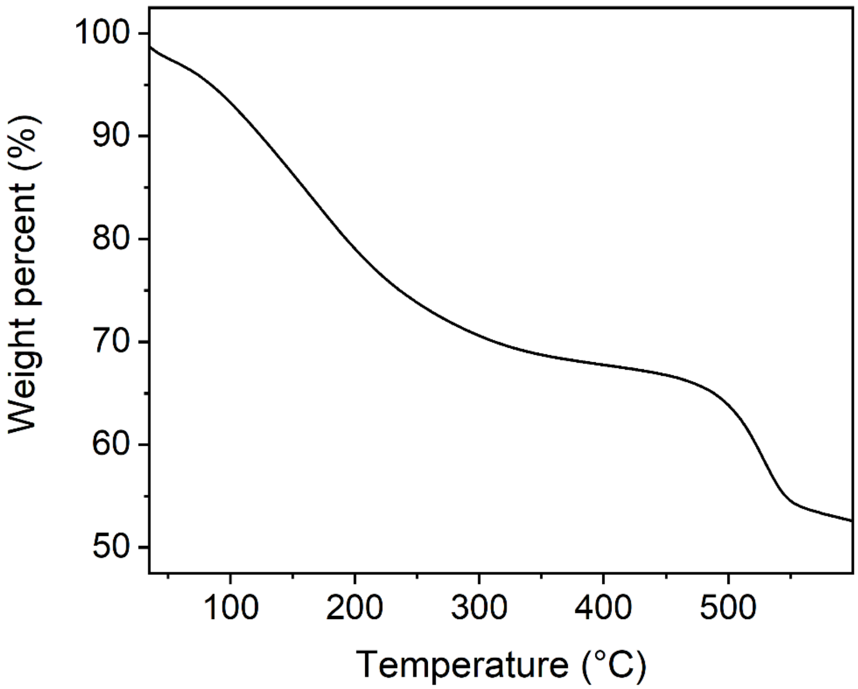

Figure S22. Thermal gravimetric analysis of $CHCl_3$-washed $Zr_{12}(\mu_3\text{-}O)_4(\mu_2\text{-}OH)_{12}(Me_2BDC)_6Cl_4$.

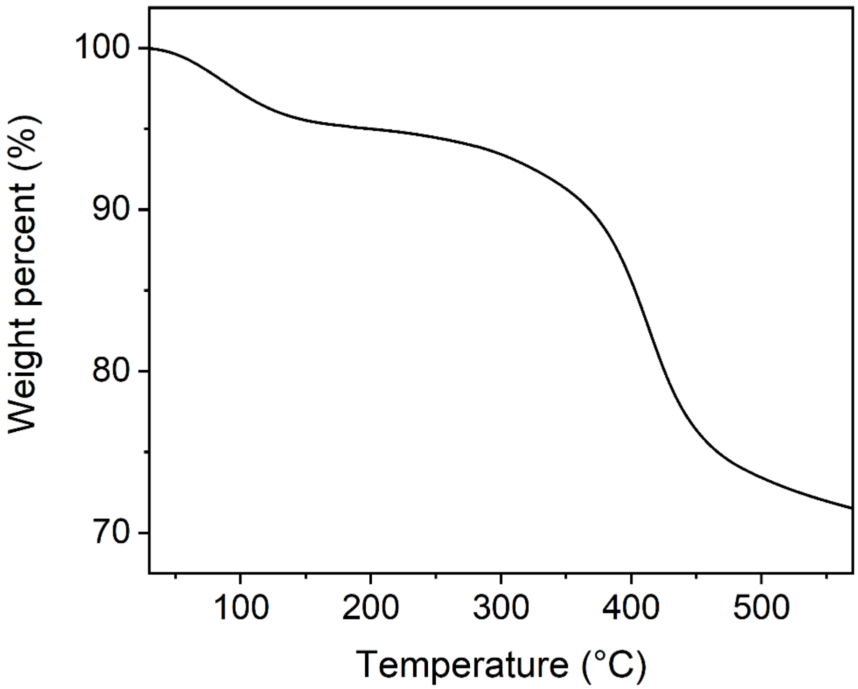

Figure S23. Thermal gravimetric analysis of $H_4Mn(tcpp)Cl$.

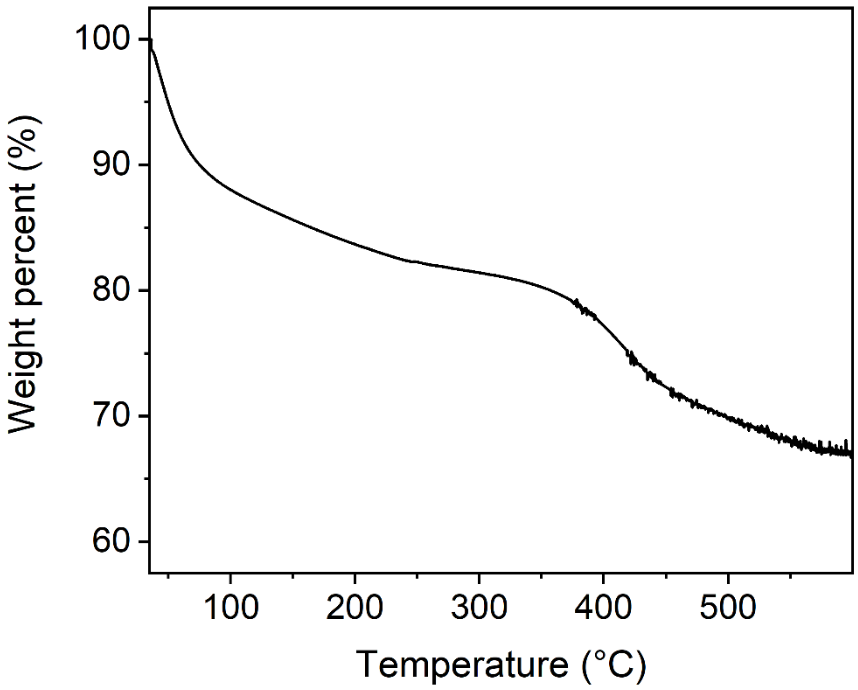

Figure S24. Thermal gravimetric analysis of methanol-washed **[ZrFDC][Mn(tcpp)Cl]**.

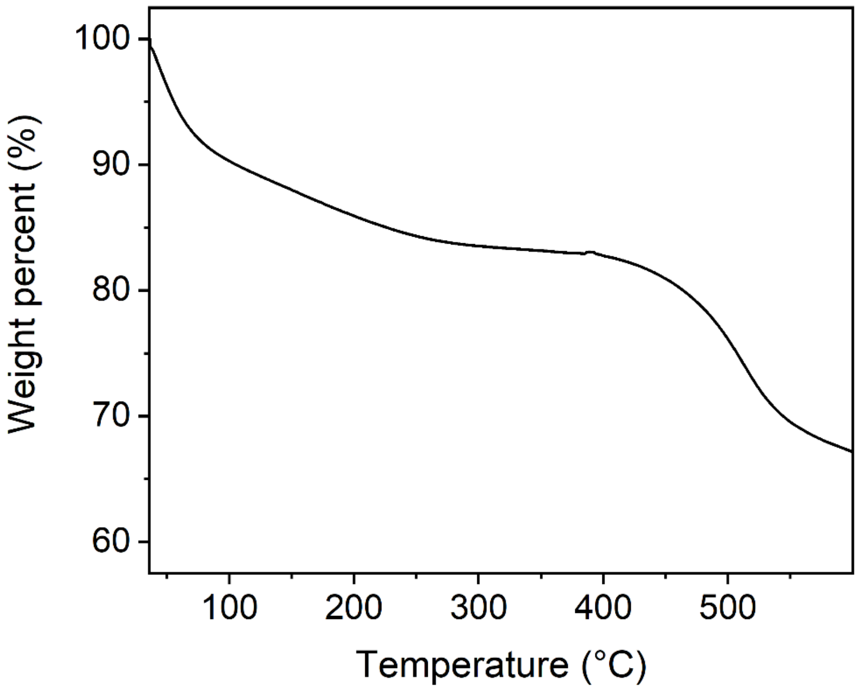

Figure S25. Thermal gravimetric analysis of methanol-washed **[$ZrMe_2BDC$][Mn(tcpp)Cl]**.

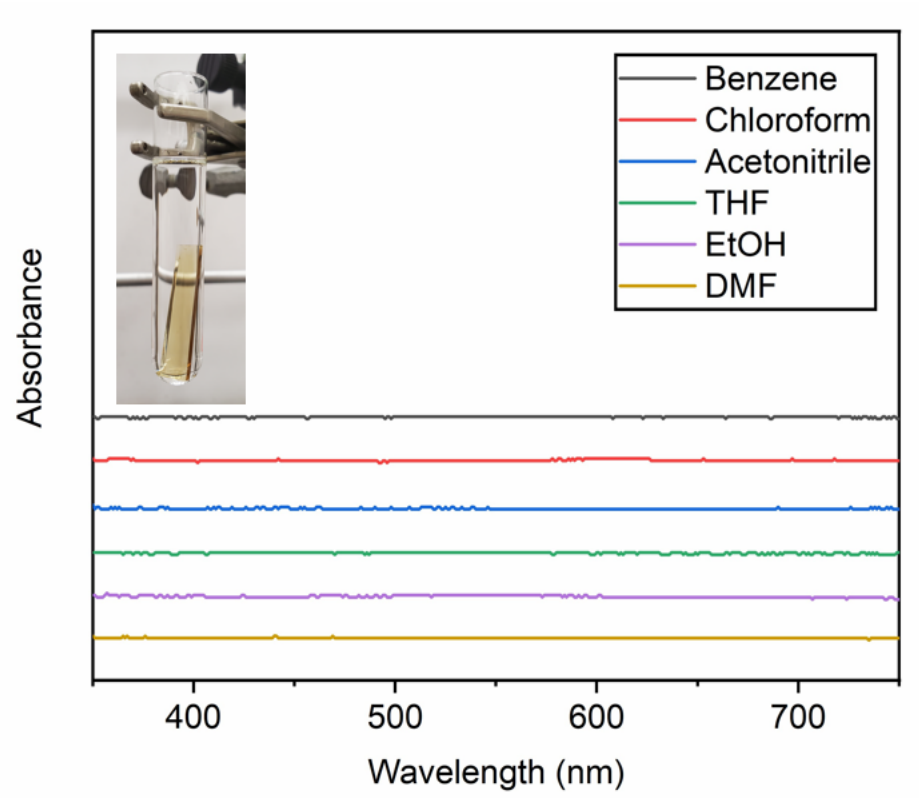

Figure S26. Transmission UV-vis spectra of various solvents decanted after soaking a thin film of **[ZrFDC][Mn(tcpp)Cl]** in 4 mL of the solvent taken in a cuvette for 30 min at 25 °C (absorbance measured with fresh solvent as the reference baseline). Inset picture showing no visible leaching of a film submerged in EtOH.

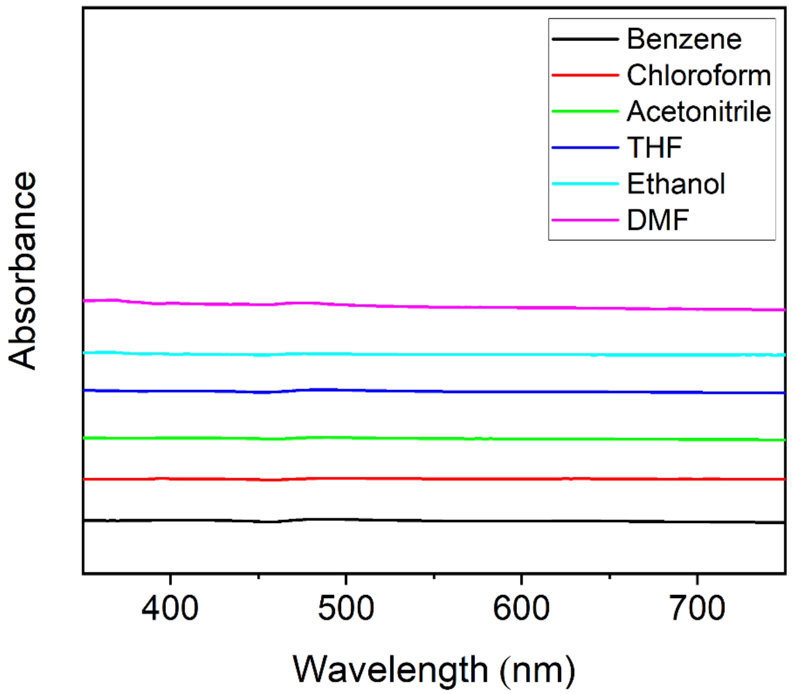

Figure S27. Transmission UV-vis spectra of various solvents decanted after soaking 10 mg of **[ZrFDC][Mn(tcpp)Cl]** salt in 4 mL of the solvent for 30 min at 25 °C (absorbance of the solvent was measured with fresh solvent as the reference baseline).

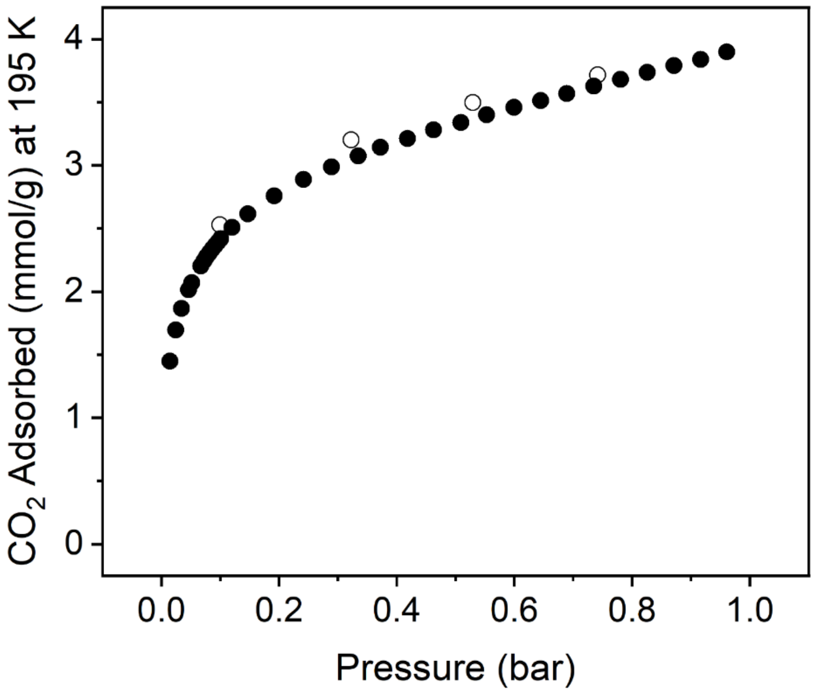

Figure S28. CO_2 adsorption isotherm for $Zr_{12}(\mu_3\text{-}O)_4(\mu_2\text{-}OH)_{12}(FDC)_6Cl_4$ that was activated at 25 °C ((adsorption (●), desorption (○)). The isotherm was measured at 195 K and provided a BET (Langmuir) surface area of 243 (441) m^2/g.

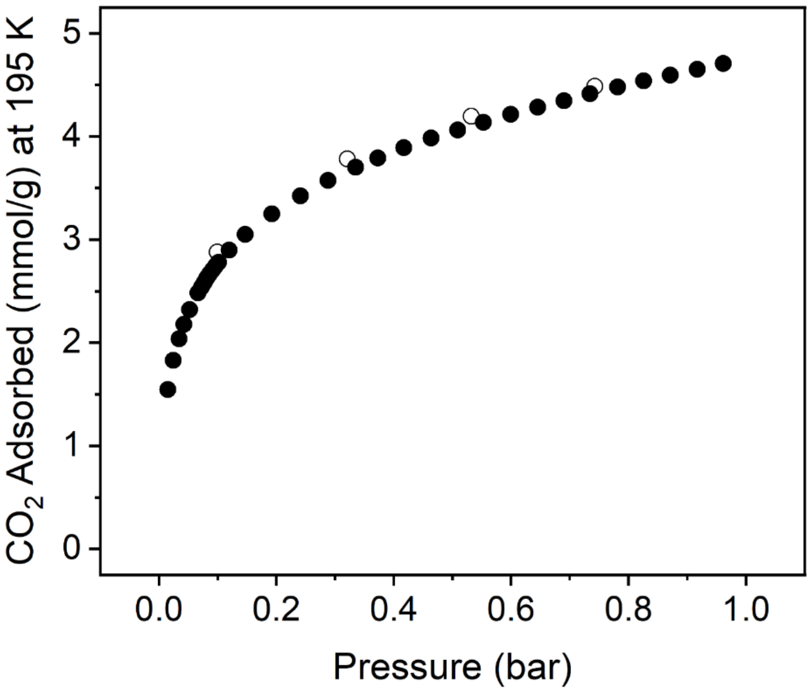

Figure S29. CO_2 adsorption isotherm for $Zr_{12}(\mu_3\text{-}O)_4(\mu_2\text{-}OH)_{12}(Me_2BDC)_6Cl_4$ that was activated at 25 °C ((adsorption (●), desorption (○)). The isotherm was measured at 195 K and provided a BET (Langmuir) surface area of 289 (549) m^2/g.

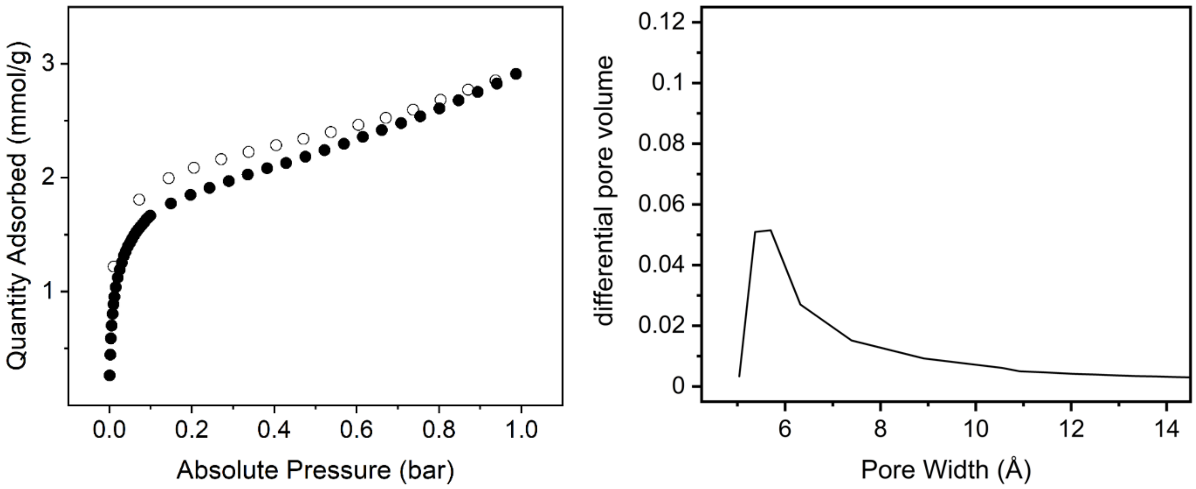

Figure S30. CO_2 adsorption isotherm for **[ZrFDC][Mn(tcpp)Cl]** that was activated at 25 °C ((adsorption (●), desorption (○)). The isotherm was measured at 195 K and provided a BET (Langmuir) surface area of 135 (244) m^2/g. An analogous N_2 isotherm at 77 K afforded the pore size distribution on the right.

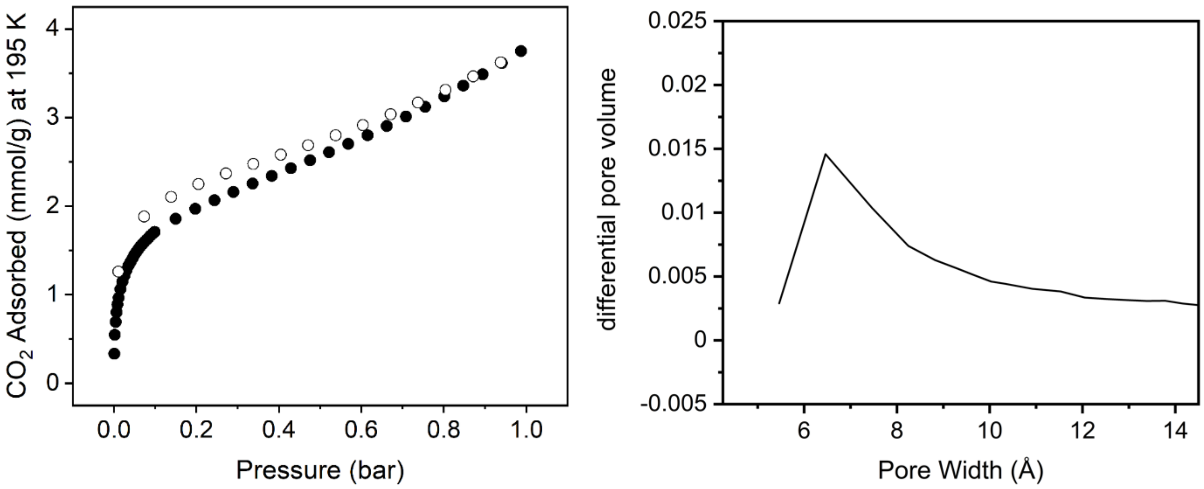

Figure S31. CO_2 adsorption isotherm for **[ZrMe$_2$BDC][Mn(tcpp)Cl]** that was activated at 25 °C ((adsorption (●), desorption (○)). The isotherm was measured at 195 K and provided a BET (Langmuir) surface area of 140 (324) m^2/g. An analogous N_2 isotherm recorded at 77 K afforded the pore size distribution on the right.

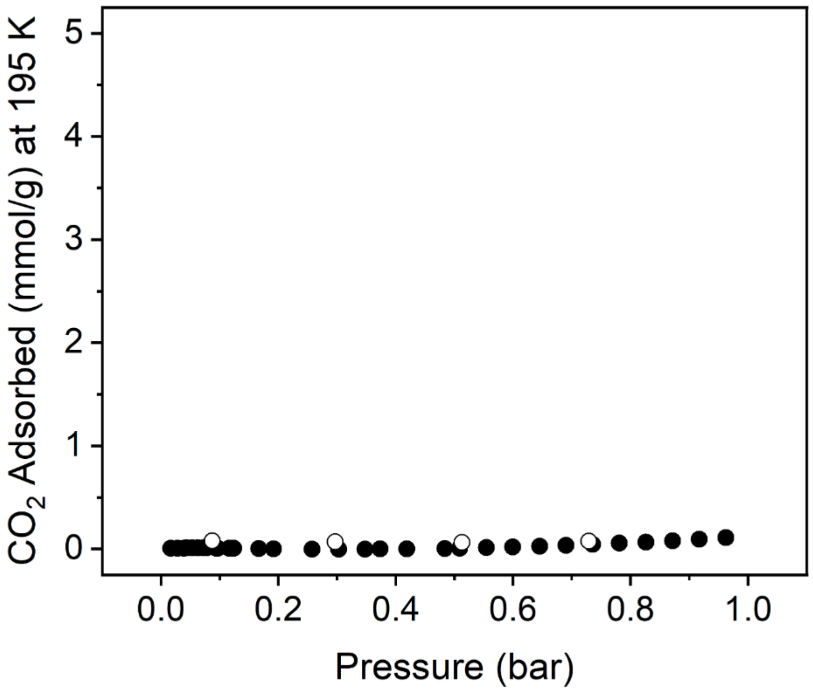

Figure S32. CO_2 adsorption isotherm for **$[HNEt_3]_4[Mn(tcpp)Cl]$** that was activated at 25 °C ((adsorption (●), desorption (○)). The isotherm was measured at 195 K and provided no measurable surface area.

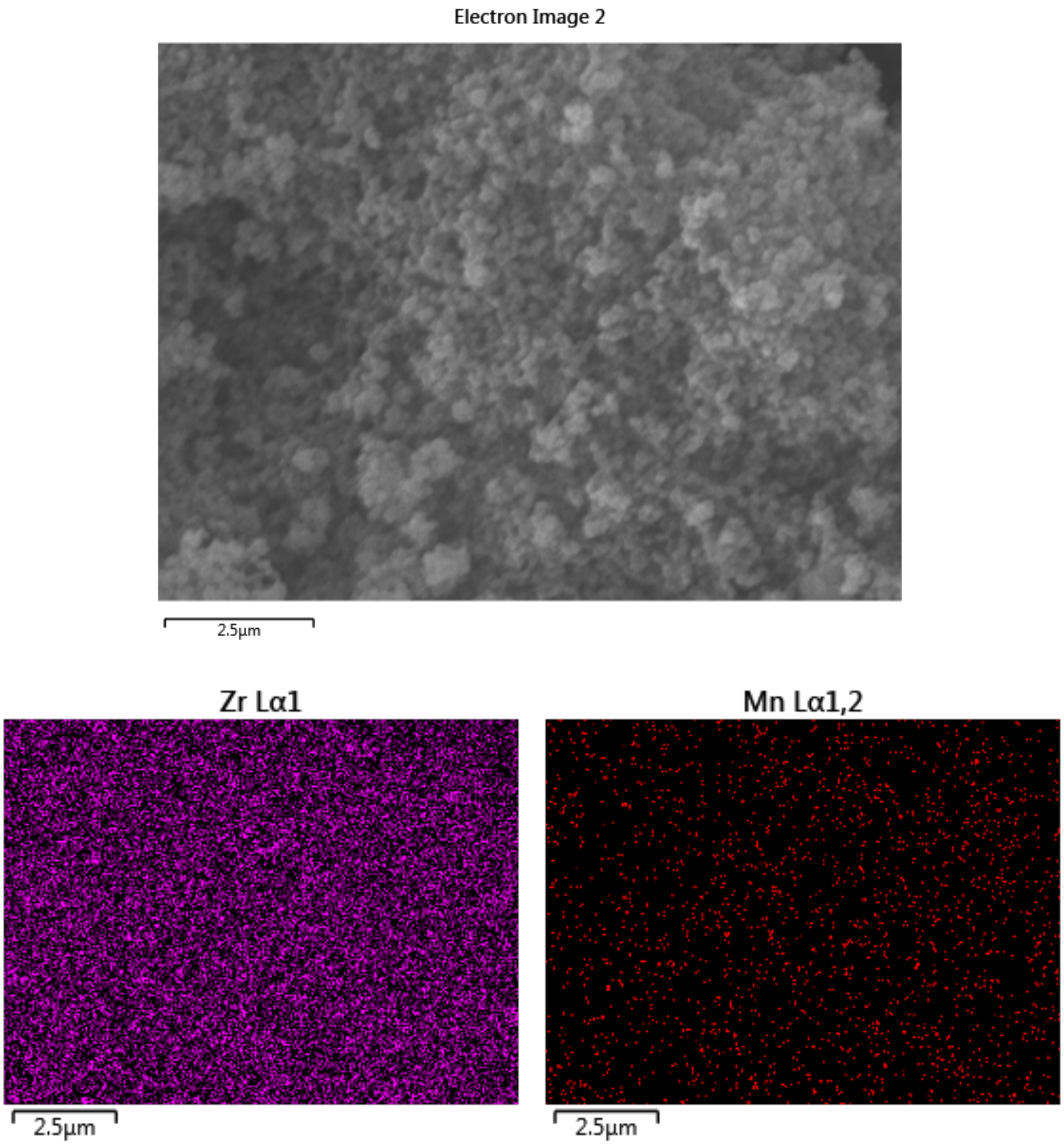

Figure S33. SEM image (top) corresponding to EDX mapping (bottom) of **[ZrFDC][Mn(tcpp)Cl]**. In the EDX map, magenta and red represent Zr (bottom left) and Mn (bottom right), respectively, showing a homogenous distribution of metals in the amorphous salt particles.

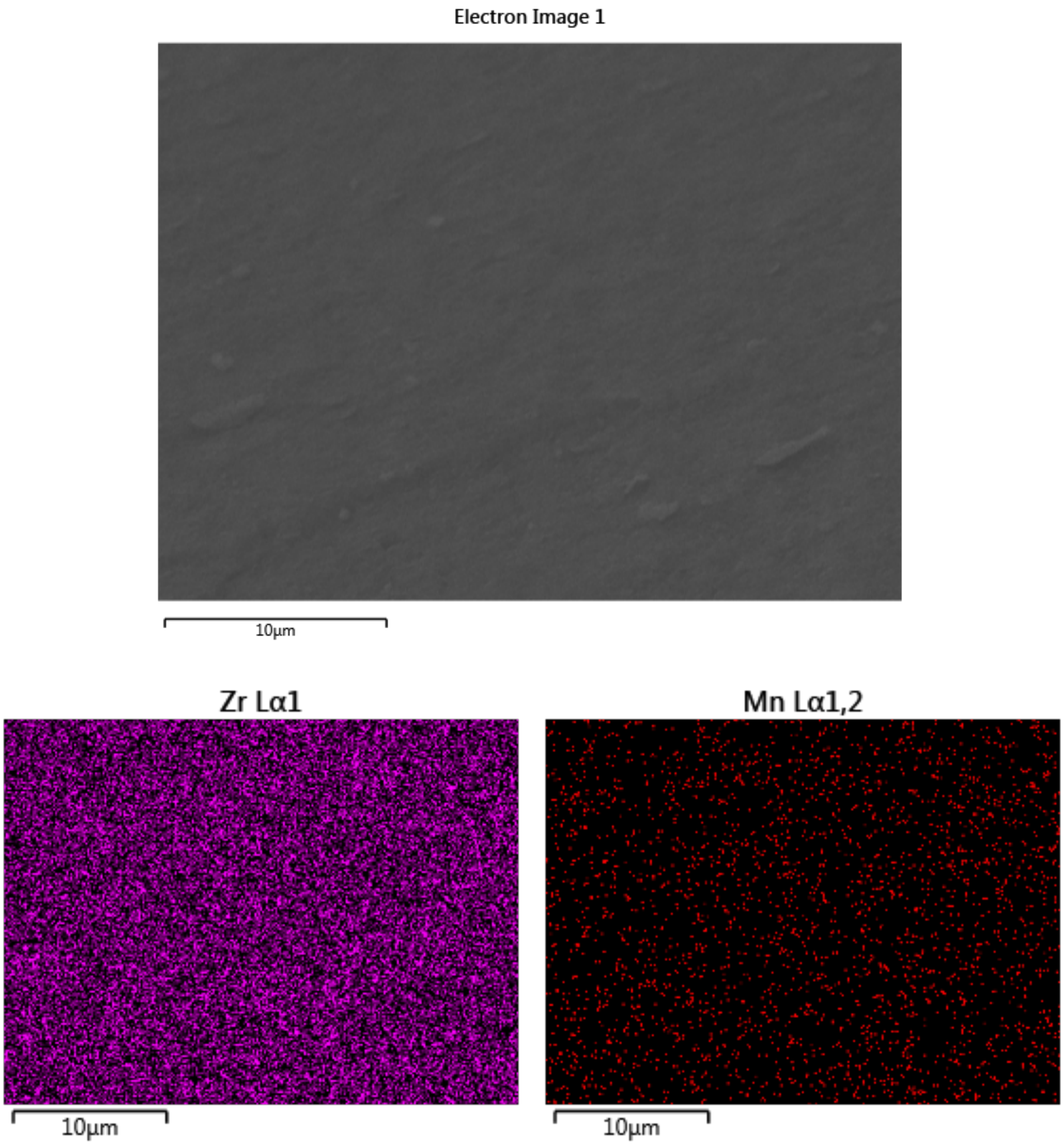

Figure S34. SEM image (top) corresponding to EDX mapping (bottom) of **[ZrMe$_2$BDC][Mn(tcpp)Cl].** In the EDX map, magenta and red represent Zr (bottom left) and Mn (bottom right), respectively, showing a homogenous distribution of metals in the amorphous salt particles.

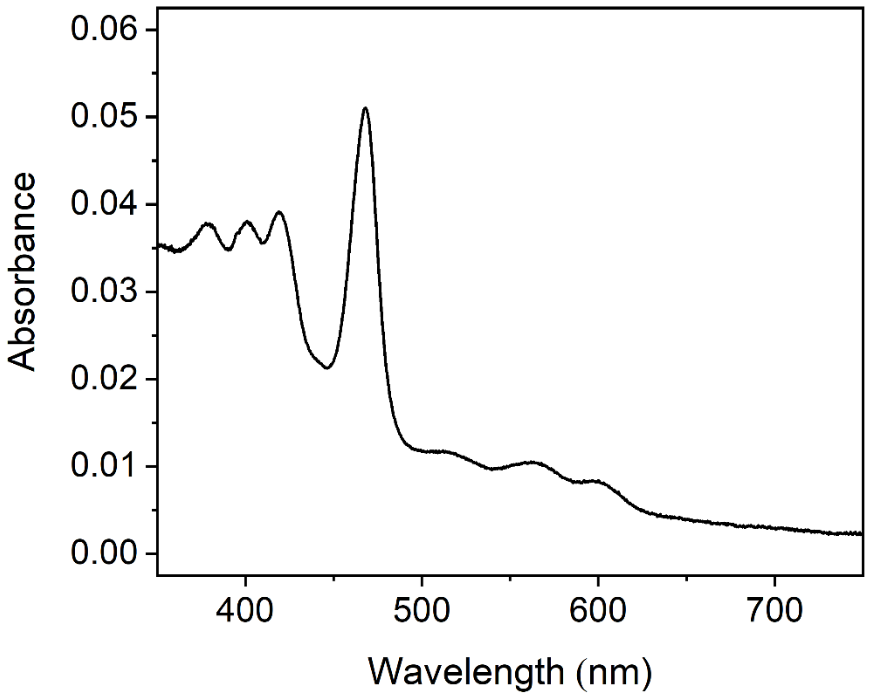

Figure S35. Transmission UV-vis spectrum of a thin film of **[ZrMe$_2$BDC][Mn(tcpp)Cl]**.

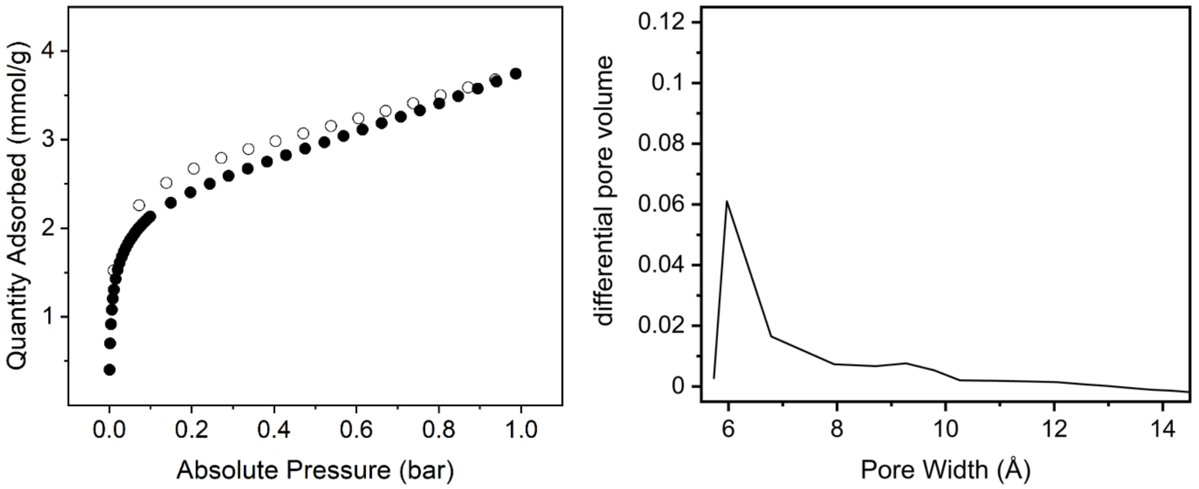

Figure S36. CO_2 adsorption isotherm for a thin film of **[ZrFDC][Mn(tcpp)Cl]** that was activated at 25 °C ((adsorption (●), desorption (○)). The isotherm was measured at 195 K and provided a BET (Langmuir) surface area of 172 (355)m^2/g. An analogous N_2 isotherm at 77 K afforded the pore size distribution on the right.

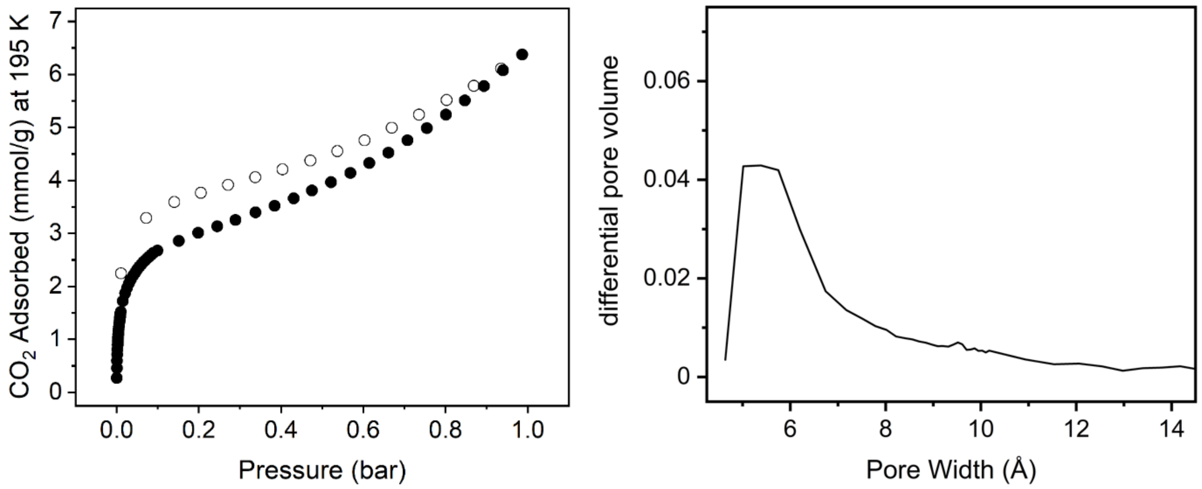

Figure S37. CO_2 adsorption isotherm for a thin film of **[ZrMe$_2$BDC][Mn(tcpp)Cl]** that was activated at 25 °C ((adsorption (●), desorption (○)). The isotherm was measured at 195 K and provided a BET (Langmuir) surface area of 214 (384) m^2/g.

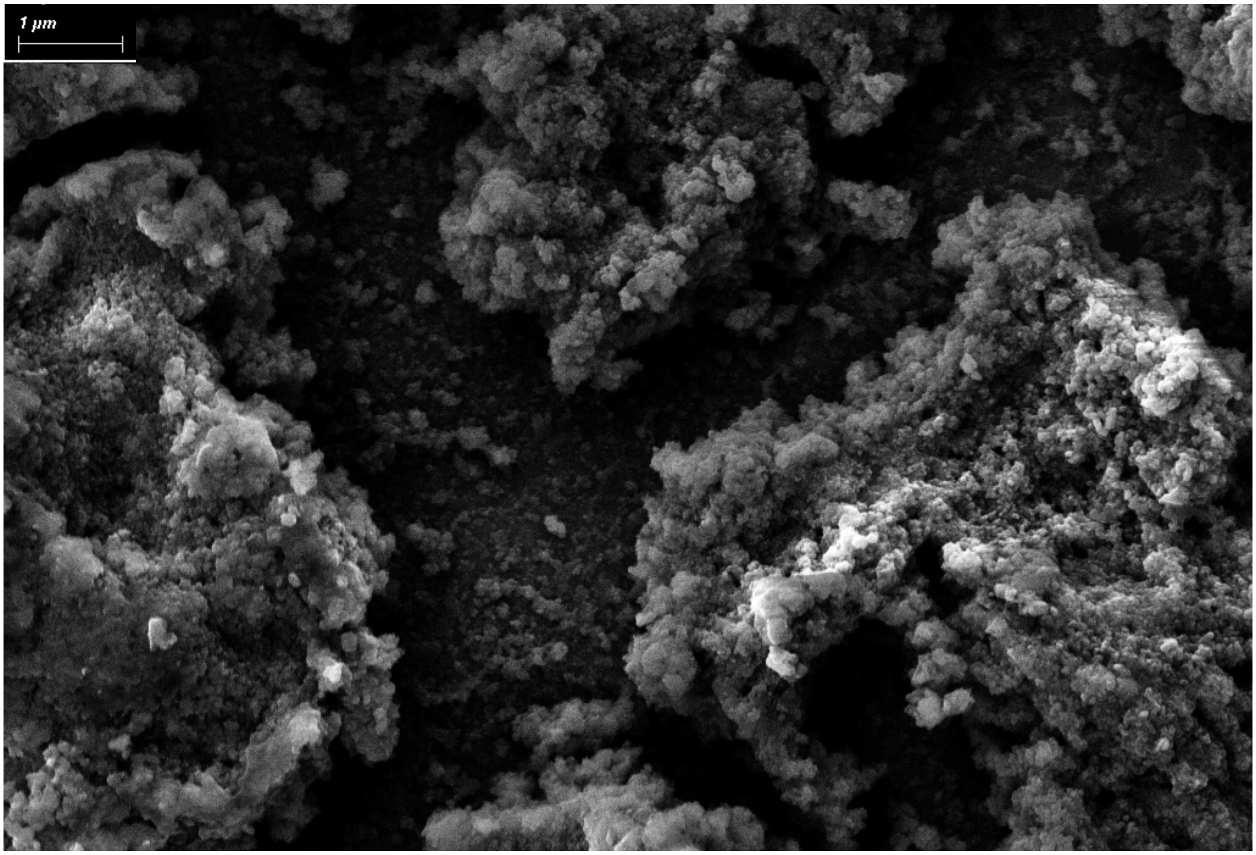

Figure S38. SEM image of a thin film of **[ZrFDC][Mn(tcpp)Cl]** on silicon.

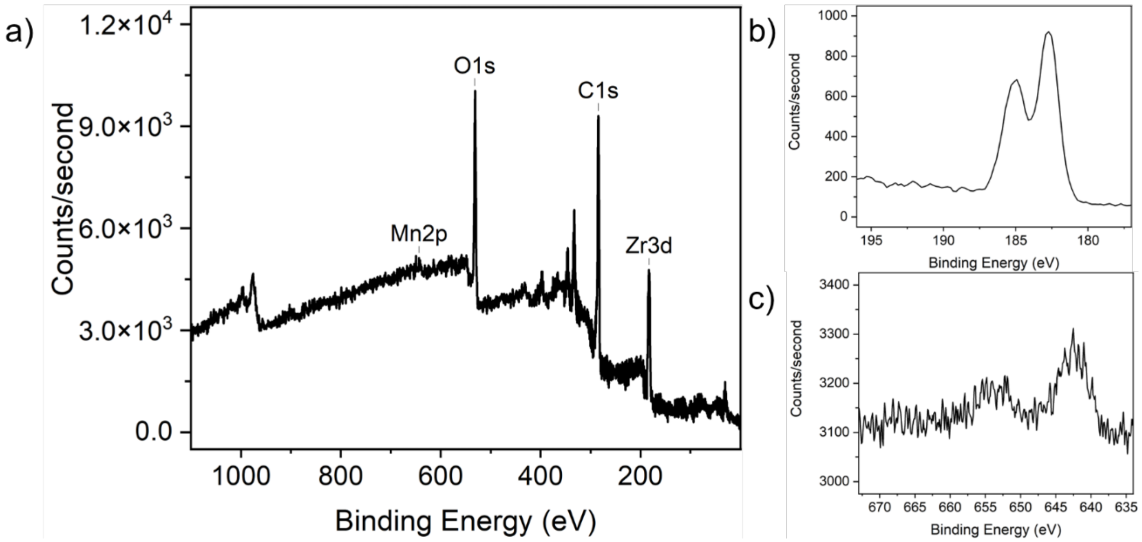

Figure S39. X-ray photoelectron spectroscopy a) survey, b) high resolution Zr3d, and c) high resolution Mn2p spectra of **[ZrFDC][Mn(tcpp)]** film.

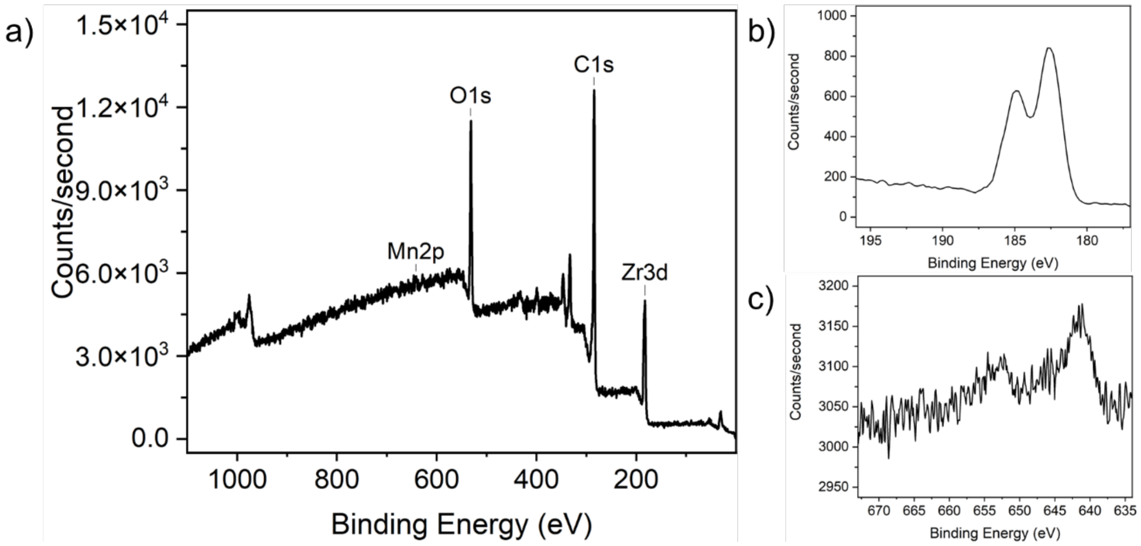

Figure S40. X-ray photoelectron spectroscopy a) survey, b) high resolution Zr3d, and c) high resolution Mn2p spectra of **[ZrMe$_2$BDC][Mn(tcpp)]** film.

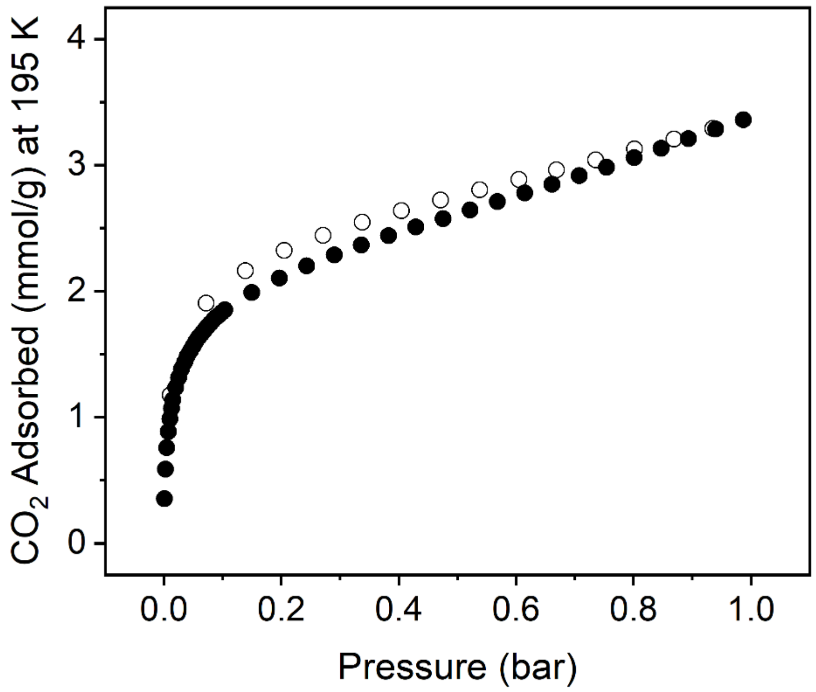

Figure S41. CO_2 adsorption isotherm for a thin film of **[ZrFDC][Mn(tcpp)]** that was activated at 25 °C ((adsorption (●), desorption (○)). The isotherm was measured at 195 K and provided a BET (Langmuir) surface area of 152 (324) m^2/g.

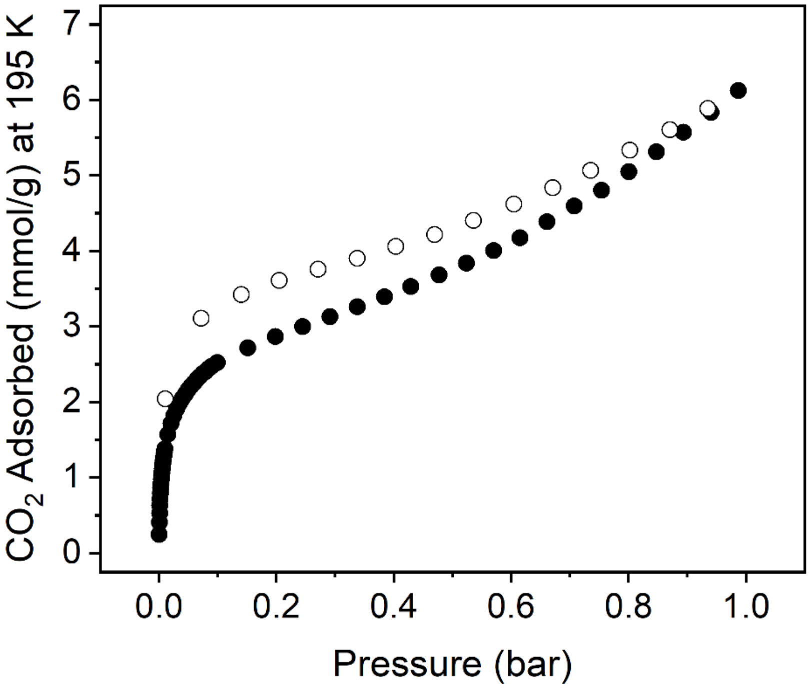

Figure S42. CO_2 adsorption isotherm for a thin film of **[ZrMe$_2$BDC][Mn(tcpp)]** that was activated at 25 °C ((adsorption (●), desorption (○)). The isotherm was measured at 195 K and provided a BET (Langmuir) surface area of 203 (360) m^2/g.

K. REFERENCES

1. Cardenal, A. D.; Jeong Park, H.; Chalker, C. J.; Ortiz, K. G.; Powers, D. C. cis-Decalin oxidation as a stereochemical probe of in-MOF versus on-MOF catalysis. *Chem. Commun.* **2017**, *53 53*, 7377–7380.
2. Zhang, W.; Jiang, P.; Wang, Y.; Zhang, J.; Zhang, P. An Efficient Catalyst Based on a Metal Metalloporphyrinic Framework for Highly Selective Oxidation. *Catal. Lett.* **2015**, *145*, 589–595.
3. Fulmer, G. R.; Miller, A. J. M.; Sherden, N. H.; Gottlieb, H. E.; Nudelman, A.; Stoltz, B. M.; Bercaw, J. E.; Goldberg, K. I. NMR Chemical Shifts of Trace Impurities: Common Laboratory Solvents, Organics, and Gases in Deuterated Solvents Relevant to the Organometallic Chemist. *Organometallics* **2010**, *29*, 2176–2179.
